# Impact of percutaneous coronary intervention with different guidance modalities in patients with coronary artery lesions: a network meta-analysis and systematic review

**DOI:** 10.3389/fcvm.2025.1526188

**Published:** 2025-10-09

**Authors:** Tao Jiang, Jie Huang, Lisha Luo, Miaoling Li, Gong Chen

**Affiliations:** ^1^ Department of Cardiology, The Affiliated Hospital of Southwest Medical University, Luzhou, Sichuan, China; ^2^ Institute of Cardiovascular Research, The Southwest Medical University, Luzhou, Sichuan, China; ^3^ General Practice, The General Hospital of Western Theater Command, Chengdu, Sichuan, China

**Keywords:** percutaneous coronary intervention, coronary artery lesions, intravascular ultrasound, optical coherence tomography, network meta-analysis

## Abstract

**Background:**

Traditional coronary angiography has inherent limitations in terms of lesion assessment and stenting. New guidance modalities to guide percutaneous coronary intervention (PCI) are now available.

**Methods:**

We systematically searched PubMed, Embase, Cochrane, and Web of Science databases for the period from the time of construction to 25 April 2024. A network meta-analysis of randomized controlled trials (RCT) was performed to determine the optimal treatment strategy by comparing the short-term outcome and long-term prognosis of adverse cardiovascular outcomes in patients with coronary artery lesions after eight different PCI-guided modalities. The clinical outcomes included major adverse cardiovascular events (MACE), all-cause mortality, cardiac death, myocardial infarction, and target vessel revascularization (TVR). Risk ratios (RR) with 95% confidence intervals (CI) were calculated.

**Results:**

Forty randomized controlled trials with a total of 38,107 patients were included. In the MACE subgroup up to 12 months, Intravascular Ultrasound-guided Percutaneous Coronary Intervention (IVUS-PCI) [RR = 1.60, 95%CI = (1.10, 2.30)], Optical Frequency Domain Imaging-guided Percutaneous Coronary Intervention (OFDI-PCI) [RR = 2.36, 95%CI = (1.05, 5.80)] and Quantitative Flow Ratio-guided Percutaneous Coronary Intervention (QFR-PCI) [RR = 1.45, 95%CI = (1.15, 1.83)] significantly reduced the incidence of MACE. In the MACE subgroup at 12 months, Fractional Flow Reserve-guided Percutaneous Coronary Intervention (FFR-PCI) [RR = 0.72, 95%CI = (0.49, 0.99)], IVUS-PCI [RR = 0.66, 95%CI = (0.43, 0.99)] and Optical Coherence Tomography-guided Percutaneous Coronary Intervention [RR = 0.59, 95%CI = (0.35, 0.92)] all significantly reduced the incidence of MACE in patients. FFR-PCI [RR = 0.42, 95%CI = (0.20, 0.75)] significantly reduced the incidence of cardiac death in patients compared to Angiography-guided Percutaneous Coronary Intervention (Angio-PCI). FFR-PCI [RR = 0.78, 95%CI = (0.62, 0.99)], OCT-PCI [RR = 0.59, 95%CI = (0.35, 0.97)], QFR-PCI [RR = 0.64, 95%CI = (0.45, 0.91)] were associated with a lower risk of myocardial infarction compared to Angio-PCI. The incidence of Target Vessel Revascularization (TVR) was significantly lower in patients who underwent IVUS-PCI [RR = 0.57, 95%CI = (0.36, 0.86)], OCT-PCI [RR = 0.47, 95%CI = (0.24, 0.95)] than in those who underwent Angio-PCI. However, there were no significant differences between the different guidance modalities and subgroup analyses in improving overall survival.

**Conclusion:**

IVUS and OCT were more effective in reducing MACE and TVR. This suggests that IVUS and OCT may be the best strategies in the interventional management of complex coronary lesions.

**Systematic Review Registration:**

PROSPERO CRD42024567598.

## 1 Introduction

Coronary artery disease (CAD) is caused by narrowed or blocked coronary arteries and is associated with extremely high global morbidity and mortality (1). In the United States alone, CAD accounts for 1 in 7 deaths, 2.2% of the total global burden of disease, and 32.7% of cardiovascular deaths, and its incidence is increasing as the population ages (2). Percutaneous coronary intervention (PCI) is widely used in the treatment of cardiovascular disease as the main means of treating coronary artery lesions (3). PCI restores blood flow by widening narrowed or blocked coronary arteries, reducing the frequency of angina attacks, lowering the risk of acute myocardial infarction, improving patients' quality of life, and prolonging life.

However, with the increasing complexity of coronary artery lesions and the emergence of individual differences in patients, it is difficult to meet the needs of precision treatment by relying on traditional angiography technology and experience for PCI. Therefore, more and more studies have explored the optimization of PCI operation and postoperative results with different guidance Modalities. Currently, imaging and physiological guidance are the two key techniques in PCI. Image-guided techniques, such as intravascular ultrasound (IVUS) and optical coherence tomography (OCT), provide high-resolution structural images of coronary arteries, helping surgeons to accurately identify lesion location, plaque nature, and stent adherence. This information can effectively improve the success rate of surgery and reduce post-operative complications. In addition, physiologically guided techniques such as fractional flow reserve (FFR) (4) and instantaneous free-wave ratio (iFR) (5) assess the impact of coronary artery stenosis on blood flow function to assist surgeons in deciding whether to intervene and evaluating postoperative treatment. Not only do these technologies help physicians make more accurate decisions in complex lesions, but they also have an important impact on post-stent optimization and long-term prognosis. The different modalities of guidance have their own advantages in clinical application. For example, studies have shown that IVUS guidance significantly reduces the incidence of in-stent restenosis and cardiovascular events (6), while FFR guidance is more helpful in avoiding unnecessary stent implantation, thereby reducing procedural risk and conserving healthcare resources while improving the quality of life for patients (7). However, due to the heterogeneity of the study populations, methods, and outcomes, there are differences in the clinical efficacy of different guidance modalities. Comparing the effectiveness of these guided approaches presents a challenge for clinicians in selecting the optimal treatment plan.

To address these challenges, network meta-analysis (NMA) is an advanced statistical tool that can provide a more comprehensive evidence base by combining direct and indirect comparisons to analyze multiple interventions simultaneously. In this study, NMA and systematic reviews were used to integrate existing clinical trials to systematically evaluate the impact of different guidance modalities on clinical outcomes in patients with coronary artery lesions in the post-operative period, including important outcome metrics such as major adverse cardiovascular events (MACE), all-cause mortality, cardiac death, myocardial infarction, and target vessel revascularization (TVR). Through this analysis, this study not only provides a scientific basis for clinicians to select the optimal guidance strategy for PCI procedures, but also provides a research direction for future improvement of PCI techniques and the long-term prognosis of patients.

## 2 Methods

Our research followed the Preferred Reporting Items for Systematic Reviews and Meta-Analyses (PRISMA) guidelines (8). Ethical approval and informed consent were not required for this study as we performed a meta-analysis of previously published data. It has been registered on the PROSPERO platform (CRD42024567598).

### 2.1 Inclusion and exclusion criteria

#### 2.1.1 Inclusion criteria

1. Population: patients with clinically diagnosed coronary artery disease, aged over 18 years.
2. Intervention/Control: Percutaneous coronary intervention (PCI) guided by CAG, CTA, IVUS, OCT, QFR, FFR, iFR, or OFDI was performed.
3. Outcome: The primary endpoint of this study was the assessment of the incidence of major adverse cardiovascular events (MACE) in the short-term or long-term (with a cut-off at 12 months of follow-up). Additionally, we assessed the incidence of all-cause mortality, cardiac death, myocardial infarction, and target vessel revascularization (TVR), which were considered secondary endpoints.
4. Study type: randomized controlled trial.

#### 2.1.2 Exclusion criteria

1. Meta-analysis, reviews, systematic reviews, expert consensus, *in vitro* studies, animal experiments, case reports, letters, responses.
2. Duplicate published literature or data
3. The literature data is incomplete or contains significant errors and attempts to contact the corresponding authors of the cited studies have been unsuccessful.

### 2.2 Search strategy

We conducted a systematic search of PubMed, Embase, Cochrane, and Web of Science databases up to April 25, 2024. The search keywords mainly include: “angiography”, “intravascular ultrasound”, “IVUS”, “optical coherence tomography”, “OCT”, “fractional flow reserve”, “FFR”, “instantaneous wave-free ratio”, “iFR”, “quantitative flow ratio”, “QFR”, “optical frequency domain imaging”, “OFDI”, “percutaneous coronary intervention”, “PCI”. In the search process, we did not set any geographical or language restrictions to ensure that the included studies were as comprehensive and representative as possible. Specific search strategies and detailed search steps for each database are described in detail in Supplementary S1.

### 2.3 Literature screening and data extraction

Two researchers (TJ and JH) conducted the literature review and data extraction strictly according to the pre-established inclusion criteria and cross-checked each other to ensure accuracy. If two researchers disagreed during the screening or extraction process, a third researcher (GC) intervened to discuss and reach an agreement through negotiation. During the data extraction process, two researchers independently extracted the following information according to the pre-established extraction list: (1) basic study characteristics, such as author, publication year, country, patient origin, age, sex, and sample size. (2) Key elements of the risk of bias assessment. (3) Outcome indicators of the study. After data extraction was completed, the two researchers cross-checked the extracted results. For the differences, the third researcher will reach the final consensus after comparing with the original literature and combining the discussion.

### 2.4 Risk of bias in the included studies

The risk of bias assessment for the included studies was independently completed by two researchers using the latest version of the Cochrane Risk of Bias tool (RoB2) to evaluate the quality of the randomized controlled trials. [https://www.riskofbias.info/welcome/rob-2-0-tool/current-version-of-rob-2/] The RoB2 tool covers five main areas of assessment, including bias in randomization; bias in departing from established interventions; bias in missing outcome data; bias in outcome measurement; and bias in selective reporting of outcomes. Each study is assessed item by item against these criteria and is classified as “low risk”, “some concern” or “high risk” based on the overall assessment results. This grading method can fully reflect the degree of research bias and ensure the rigour and consistency of the quality assessment of the included literature. If there is a disagreement between two reviewers in the assessment process, a third reviewer will intervene and reach a consensus through negotiation.

### 2.5 Statistical analysis

All analyses were performed with Stata 15.1 and R software (version 4.0.3). We used risk ratios (RR) and their 95% confidence intervals (CI) for the analysis of dichotomous variables. Network meta-analysis can use either a random effects model or a fixed effects model. The random effects model was chosen based on the assumption that the true effect sizes may differ between studies, considering the clinical and methodological heterogeneity in the included trials. In contrast, the fixed effects model assumes that the true effect size is the same across all studies. To account for possible heterogeneity between experiments, we used a Bayesian random-effects model to analyze the treatment effect of PCI under different guidance methods in patients with coronary artery disease. In the modeling process, the Markov chain Monte Carlo (MCMC) method was used to run four Markov chains, and the number of annealing times was set to 20,000. After 50,000 simulation iterations, the model was established and optimized. The Deviance Information Criterion (DIC) was used to assess the fit and global consistency of the model. DIC is a criterion used for evaluating Bayesian models, which balances model fit and complexity. A lower DIC value indicates a better model. If there are closed loops in the network structure, we will use the node-splitting method to analyze the local consistency further. In addition, we will rank each intervention based on the area under the cumulative ranking curve (SUCRA) and create a league table to compare the differences in effect between each intervention. SUCRA is a statistical method used in network meta-analysis, which calculates the area under the cumulative ranking curve for each intervention method to derive the ranking probability of the interventions. The SUCRA value ranges from 0 to 1, with higher values indicating better performance of the intervention in all comparisons. We used *p*-values to assess the statistical significance of the results, with *p* < 0.05 indicating a statistically significant difference. We will plot funnel plots to visually represent the heterogeneity between trials.

## 3 Results

### 3.1 Systematic search results

**Figure 1:**
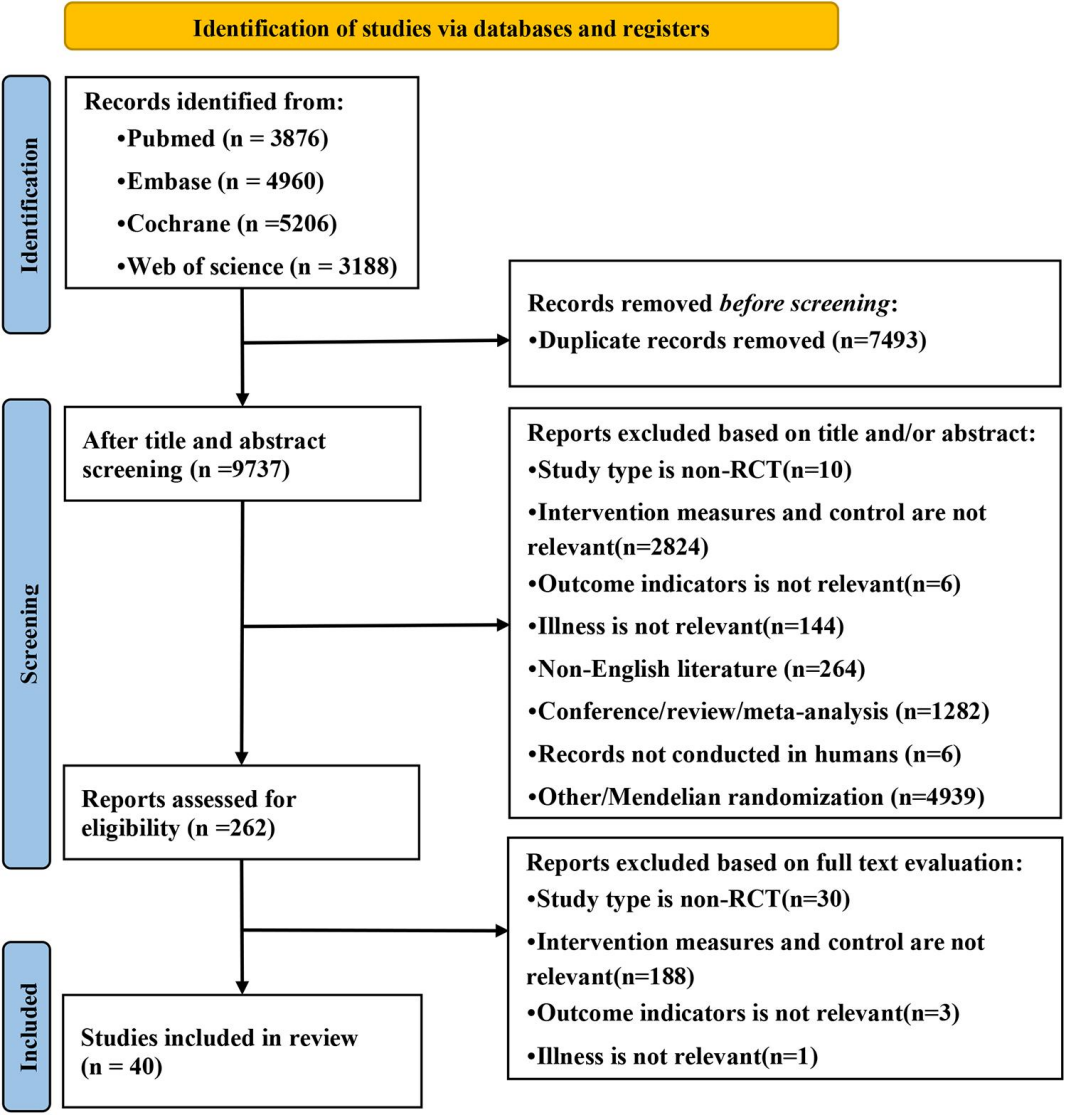
PRISMA flow diagram of the search for relevant trials.

A total of 17,230 articles were retrieved from the database. After deduplication, 7,493 duplicates were removed and 9,737 independent studies were retained. Then, the titles and abstracts were screened to exclude 9,475 unrelated articles, and the remaining 262 articles were analyzed for full-text reading. According to the exclusion criteria, 222 ineligible studies were deleted and 40 randomized controlled trials were included (7, 9–47). The research selection process is shown in Figure 1.

### 3.2 Characteristics of included studies

**Figure 2:**
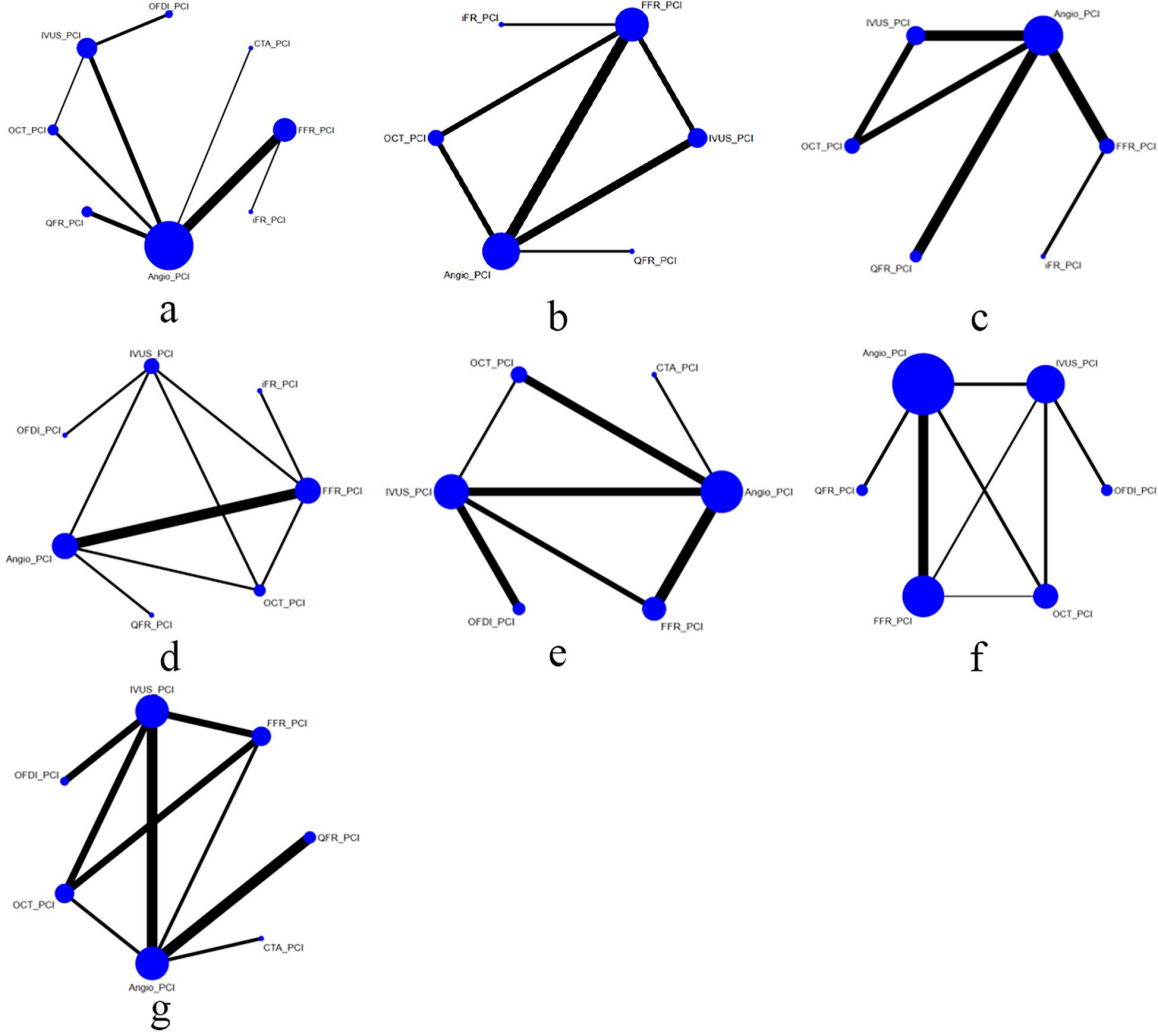
Comparisons of clinical outcomes among guidance modalities included in the network meta-analysis. **(a)** MACE ≤12 months; **(b)** MACE >12 months; **(c)** all-cause mortality ≤12 months; **(d)** all-cause mortality >12 months; **(e)** cardiac death; **(f)** myocardial infarction; **(g)** TVR.

Of the included trials, eleven studies compared FFR with CAG (11, 13, 18, 25, 29, 31, 33, 35, 36, 38, 46), six trials compared IVUS with CAG (20, 28, 34, 41, 43, 47), and six trials compared OCT with CAG (12, 24, 30, 32, 42, 45). Four trials assessed differences between QFR and CAG (10, 15, 16, 26), one study assessed differences between CTA and CAG (37), and two trials each compared FFR and IVUS (9, 17), OCT and IVUS (7, 14), iFR and FFR (19, 44), and FFR and OCT (22, 39). Three trials compared OFDI with IVUS (21, 23, 27), and one trial compared OCT, IVUS, and CAG (40). The baseline characteristics of the trial and participants are shown in Table 1. Figure 2 shows a network diagram of outcomes for different interventions.

**Table 1:** Main characteristics of the trials.

Trial	Comparison	Region	Sample size	Male/Female	Age (years)	Follow-up (month)	Outcomes
Koo 2022 (9)	FFR vs. IVUS	Korea China	1,682 (838 vs. 844)	1,187 vs. 493	65.10 ± 9.60	24	MACE, ACM, CD, MI, TVR
Xu 2021 (10)	QFR vs. CAG	China	3,825 (1,913 vs. 1,912)	2,699 vs. 1,126	62.70 ± 10.10	12	MACE, ACM, MI, TVR
Lee 2023 (11)	FFR vs. CAG	Korea	562 (284 vs. 278)	474 vs. 88	63.30 ± 11.40	48	MACE, ACM, CD, MI
Holm 2023 (12)	OCT vs. CAG	Europe	1,201 (600 vs. 601)	948 vs. 253	66.30 ± 10.20	48	MACE, ACM, CD
Tonino 2009 (13)	FFR vs. CAG	United States Europe	1,005 (509 vs. 496)	744 vs. 261	64.60 ± 10.30	12	MACE, MI
Kang 2023 (14)	OCT vs. IVUS	South Korea	2,008 (1,005 vs. 1,003)	1,575 vs. 433	64.30 ± 10.30	12	ACM, CD, MI, TVR
Song 2022 (15)	QFR vs. CAG	China	3,825 (1,913 vs. 1,912)	–	62.70	24	MACE, ACM, MI, TVR
Kang 2024 (7)	OCT vs. IVUS	South Korea	1,475 (719 vs. 756)	1,162 vs. 313	64.80 ± 10.10	36	ACM, CD, MI
Zhang 2022 (16)	QFR vs. CAG	China	3,221 (1,669 vs. 1,552)	–	–	12	MACE, ACM, MI, TVR
Lee 2023 (17)	FFR vs. IVUS	Korea China	831 (305 vs. 526)	560 vs. 271	65.10 ± 9.60	24	MACE, CD, MI, TVR
Rioufol 2021 (18)	FFR vs. CAG	France	927 (460 vs. 467)	778 vs. 149	65.00 ± 10.00	12	MACE, ACM, MI
Andell 2018 (19)	iFR vs. FFR	Sweden Denmark Iceland	814 (405 vs. 409)	605 vs. 209	66.80 ± 8.60	12	MACE, ACM
Tan 2015 (20)	IVUS vs. CAG	China	123 (61 vs. 62)	81 vs. 42	75.85 ± 3.49	36	MACE
Kubo 2017 (21)	OFDI vs. IVUS	Japan	817 (412 vs. 405)	637 vs. 180	69.00 ± 9.00	12	MACE, CD, MI, MI
Burzotta 2020 (22)	FFR vs. OCT	Italy	350 (176 vs. 174)	261 vs. 89	68.00 ± 10.00	13	MACE, ACM, TVR
Otake 2018 (23)	OFDI vs. IVUS	Japan	103 (54 vs. 49)	84 vs. 19	68.00 ± 8.00	8	MACE, CD, MI, MI, TVR
Ueki 2020 (24)	OCT vs. CAG	European	38 (19 vs. 19)	30 vs. 8	63.30 ± 12.70	12	MI, TVR
Fearon 2010 (25)	FFR vs. CAG	United States	1,005 (509 vs. 496)	744 vs. 261	64.60 ± 10.30	12	MACE, MI
Wang 2023 (26)	QFR vs. CAG	China	2,205 (814 vs. 1,391)	1,540 vs. 665	60.20 ± 10.60	12	MACE, ACM, MI
Muramatsu 2020 (27)	OFDI vs. IVUS	Japan	109 (54 vs. 55)	85 vs. 24	72.00 ± 2.75	36	ACM, CD, MI, MI, TVR
Gaster 2001 (28)	IVUS vs. CAG	Denmark	108 (54 vs. 54)	–	57.00 ± 11.25	6	TVR
Nunen 2015 (29)	FFR vs. CAG	European United States	865 (439 vs. 429)	646 vs. 219	64.50 ± 10.40	60	MACE, ACM, CD, MI
Kala 2018 (30)	OCT vs. CAG	Czech	201 (105 vs. 96)	170 vs. 31	57.00 ± 6.00	9	MACE
Zhang 2016 (31)	FFR vs. CAG	China	220 (110 vs. 110)	153 vs. 67	70.00 ± 3.70	12	MACE, ACM
Meneveau 2016 (32)	OCT vs. CAG	France	240 (120 vs. 120)	186 vs. 54	60.50 ± 11.40	6	ACM, MI, TVR
Pijls 2010 (33)	FFR vs. CAG	European United States	1,005 (509 vs. 496)	744 vs. 261	64.60 ± 10.30	24	MI
Gaster 2003 (34)	IVUS vs. CAG	European United States	108 (54 vs. 54)	–	57.00 ± 8.25	48	MACE
Seung 2024 (35)	FFR vs. CAG	Korea	184 (103 vs. 81)	157 vs. 27	61.80 ± 10.60	48	MACE, ACM, CD, MI
Layland 2015 (36)	FFR vs. CAG	UK	350 (176 vs. 174)	260 vs. 90	62.30 ± 11.00	12	MACE, ACM
Hong 2021 (37)	CTA vs. CAG	South Korea	400 (200 vs. 200)	326 vs. 74	62.00 ± 10.00	12	MACE, CD, TVR
Puymirat 2023 (38)	FFR vs. CAG	France	1,163 (586 vs. 577)	966 vs. 197	62.20 ± 11.20	36	MACE, ACM, MI
Leone 2022 (39)	FFR vs. OCT	Italy	201 (119 vs. 82)	146 vs. 55	68.00 ± 90.00	24	MACE, MI, TVR
Ali 2021 (40)	OCT vs. IVUS vs. CAG	United States	431 (153 vs. 136 vs. 142)	323 vs. 108	66.00 ± 3.25	12	MACE, ACM, MI
Hong 2015 (41)	IVUS vs. CAG	Korea	1,400 (700 vs. 700)	964 vs. 436	64.00 ± 9.00	12	MACE, CD
Jia 2022 (42)	OCT vs. CAG	China	226 (112 vs. 114)	180 vs. 46	55.50 ± 10.80	12	CD
Zhang 2018 (43)	IVUS vs. CAG	China	1,448 (724 vs. 724)	1,065 vs. 383	65.20 ± 10.90	12	ACM, CD, MI
Berntorp 2023 (44)	iFR vs. FFR	Sweden	908 (473 vs. 435)	616 vs. 292	67.40 ± 9.70	60	MACE, ACM
Ali 2024 (45)	OCT vs. CAG	United States	1,973 (992 vs. 981)	1,542 vs. 431	65.60 ± 10.50	24	MACE, CD, MI
Chen 2015 (46)	FFR vs. CAG	China	320 (160 vs. 160)	237 vs. 83	65.20 ± 9.60	12	MACE, CD, MI, TVR
Tian 2015 (47)	IVUS vs. CAG	China	230 (115 vs. 115)	–	–	24	MACE, ACM, CD, MI

MACE, major adverse cardiovascular events; ACM, all-cause mortality; CD, cardiac death; MI, myocardial infarction; TVR, target vessel revascularization; CAG, coronary angiography.

### 3.3 Risk assessment of bias

We assessed the quality of the included trials using the Cochrane Recommendation Assessment Tool ROB2. Outcome data were described in detail in 40 trials. 33 described random components of the sequence generation process, such as computer-generated random numbers or random number tables. The high risk comes mainly from the measurement of outcomes. As Supplementary Material S2.

### 3.4 Primary outcomes

#### 3.4.1 MACE

In 17 trials (10, 13, 16, 18, 19, 21, 23, 25, 26, 30, 31, 36, 37, 40, 41, 46, 47), the occurrence of major adverse cardiovascular events (MACE) within 12 months was reported. The results of the network meta-analysis showed IVUS-PCI [RR = 1.60, 95%CI = (1.10, 2.30)], OFDI-PCI [RR = 2.36, 95%CI = (1.05, 5.80)], QFR-PCI [RR = 1.45, 95%CI = (1.15, 1.83)] compared with Angio-PCI, the incidence of MACE was significantly reduced in patients. In contrast, CTA-PCI [RR = 1.25, 95%CI = (0.47, 3.37)], FFR-PCI [RR = 1.21, 95%CI = (0.98, 1.47)], iFR-PCI [RR = 1.06, 95%CI = (0.59, 1.88)] and OCT-PCI [RR = 0.94, 95%CI = (0.50, 1.79)] did not show statistically significant differences. Detailed results can be found in Table 2 (MACE league table—bottom left).

**Table 2:** League table of MACE.

Angio-PCI	–	0.72 (0.49, 0.99)	0.79 (0.33, 1.85)	0.66 (0.43, 0.99)	0.59 (0.35, 0.92)	–	0.68 (0.31, 1.47)
1.25 (0.47, 3.37)	CTA-PCI	–	–	–	–	–	–
1.21 (0.98, 1.47)	0.96 (0.35, 2.60)	FFR-PCI	1.11 (0.50, 2.46)	0.92 (0.59, 1.46)	0.82 (0.48, 1.35)	–	0.94 (0.42, 2.26)
1.06 (0.59, 1.88)	0.85 (0.26, 2.59)	0.88 (0.51, 1.50)	iFR-PCI	0.83 (0.34, 2.10)	0.74 (0.28, 1.87)	–	0.85 (0.27, 2.79)
1.60 (1.10, 2.30)	1.29 (0.44, 3.59)	1.33 (0.87, 2.01)	1.51 (0.76, 2.97)	IVUS-PCI	0.89 (0.47, 1.61)	–	1.03 (0.43, 2.51)
0.94 (0.50, 1.79)	0.75 (0.23, 2.36)	0.78 (0.40, 1.51)	0.89 (0.38, 2.10)	0.59 (0.30, 1.16)	OCT-PCI	–	1.15 (0.48, 3.00)
2.36 (1.05, 5.80)	1.87 (0.51, 7.12)	1.95 (0.85, 4.94)	2.22 (0.84, 6.40)	1.47 (0.70, 3.33)	2.51 (0.92, 7.26)	OFDI-PCI	–
1.45 (1.15, 1.83)	1.16 (0.42, 3.18)	1.20 (0.89, 1.65)	1.36 (0.74, 2.57)	0.90 (0.60, 1.41)	1.54 (0.78, 3.05)	0.61 (0.24, 1.43)	QFR-PCI

Lower left corner, ≤12 months; Top right, >12 months.

In 15 trials (9, 11, 12, 15, 17, 20, 22, 29, 34, 35, 38, 39, 44, 45, 47), the occurrence of MACE after 12 months was reported. The results of the network meta-analysis showed that FFR-PCI [RR = 0.72, 95%CI = (0.49, 0.99)], IVUS-PCI [RR = 0.66, 95%CI = (0.43, 0.99)] and OCT-PCI [RR = 0.59, 95%CI = 0.35, 0.92)] compared with Angio-PCI, the incidence of MACE was significantly reduced. However, iFR-PCI [RR = 0.79, 95%CI = (0.33, 1.85)] and QFR-PCI [RR = 0.68, 95%CI = (0.31, 1.47)] did not show statistically significant differences compared with Angio-PCI. Detailed results are shown in Table 2 (MACE league table—top right corner).

#### 3.4.2 All-cause mortality

In 12 trials (10, 14, 16, 18, 19, 26, 31, 32, 36, 40, 43, 47), the occurrence of all-cause mortality within 12 months was reported. The results of network meta-analysis showed that Angio-PCI was the experimental group, FFR-PCI [RR = 0.68, 95%CI = (0.32, 1.41)], iFR-PCI [RR = 0.55, 95%CI = (0.12, 2.35)], IVUS-PCI [RR = 1.32, 95%CI = (0.57, 2.91)], OCT-PCI [RR = 1.67, 95%CI = (0.49, 5.45)] and QFR-PCI [RR = 0.73, 95%CI = (0.34, 1.43)] did not showed significant difference in all-cause mortality. Detailed results are shown in Table 3 (bottom left corner of All-cause mortality league table).

**Table 3:** League table of All-cause mortality.

Angio-PCI	0.58 (0.24, 1.07)	0.67 (0.11, 3.06)	0.77 (0.24, 2.12)	0.64 (0.19, 1.74)	0.95 (0.21, 4.44)	0.99 (0.11, 8.53)
0.68 (0.32, 1.41)	FFR-PCI	1.14 (0.26, 5.09)	1.32 (0.48, 4.14)	1.11 (0.36, 3.55)	1.62 (0.34, 10.34)	1.73 (0.21, 16.29)
0.55 (0.12, 2.35)	0.81 (0.22, 2.87)	iFR-PCI	1.15 (0.20, 7.73)	0.96 (0.15, 6.44)	1.41 (0.17, 15.94)	1.50 (0.12, 21.98)
1.32 (0.57, 2.91)	1.93 (0.64, 5.75)	2.40 (0.44, 12.85)	IVUS-PCI	0.84 (0.26, 2.51)	1.23 (0.20, 8.70)	1.29 (0.20, 8.83)
1.67 (0.49, 5.45)	2.47 (0.58, 9.86)	3.07 (0.45, 20.04)	1.28 (0.45, 3.52)	OCT-PCI	1.47 (0.25, 10.95)	1.55 (0.18, 14.80)
0.73 (0.34, 1.43)	1.08 (0.37, 2.94)	1.34 (0.25, 6.84)	0.55 (0.19, 1.62)	0.44 (0.11, 1.74)	QFR-PCI	1.05 (0.07, 14.26)
–	–	–	–	–	–	OFDI-PCI

Lower left corner, ≤12months; Top right, >12months.

In 12 trials (7, 9, 11, 12, 15, 22, 27, 29, 35, 38, 44, 47), the occurrence of all-cause mortality after 12 months was reported. The results of network meta-analysis showed that FFR-PCI [RR = 0.58, 95%CI = (0.24, 1.07)], iFR-PCI [RR = 0.67, 95%CI = (0.11, 3.06)], IVUS-PCI [RR = 0.77, 95%CI = (0.24, 2.12)], OCT-PCI [RR = 0.64, 95%CI = (0.19, 1.74)], QFR-PCI [RR = 0.95 95%CI = (0.21, 4.44)], OFDI-PCI [RR = 0.99, 95%CI = (0.11, 8.53)] The incidence of all-cause mortality was not statistically significantly different from Angio-PCI. Detailed results are shown in Table 3 (All-cause mortality league table—top right corner).

#### 3.4.3 Cardiac death

In 17 trials (9, 11, 12, 14, 17, 21, 23, 27, 29, 35, 37, 41–43, 45–47), the occurrence of cardiac death was reported. The results of the network meta-analysis showed FFR-PCI [RR = 0.42, 95%CI = (0.20, 0.75)] compared with Angio-PCI, the incidence of cardiac death was significantly reduced in patients. However, CTA-PCI [RR = 2.46, 95%CI = (0.16, 83.51)], IVUS-PCI [RR = 0.68, 95%CI = (0.34, 1.33)], OCT-PCI [RR = 0.5, 95%CI = (0.24, 1.02)], OFDI-PCI [RR = 0.67, 95%CI = (0.11, 3.54)] did not show statistically significant differences compared with Angio-PCI. Detailed results are shown in Table 4.

**Table 4:** League table of cardiac death.

Angio-PCI	2.46 (0.16, 83.51)	0.42 (0.2, 0.75)	0.68 (0.34, 1.33)	0.5 (0.24, 1.02)	0.67 (0.11, 3.54)
	CTA-PCI	0.17 (0.01, 2.81)	0.28 (0.01, 4.54)	0.2 (0.01, 3.58)	0.27 (0.01, 6.56)
		FFR-PCI	1.62 (0.78, 3.76)	1.19 (0.49, 3.26)	1.6 (0.27, 9.11)
			IVUS-PCI	0.74 (0.3, 1.81)	0.99 (0.19, 4.57)
				OCT-PCI	1.33 (0.21, 7.97)
					OFDI-PCI

#### 3.4.4 Myocardial infarction

In 25 trials (7, 9, 11, 13, 15–18, 21, 23–27, 29, 32, 33, 35, 38–40, 43, 45–47), the occurrence of myocardial infarction was reported. The results of network meta-analysis demonstrated that the use of FFR-PCI [RR = 0.78, 95%CI = (0.62, 0.99)], QCT-PCI [RR = 0.59, 95%CI = (0.35, 0.97)], QFR-PCI [RR = 0.64, 95%CI = (0.45, 0.91)] was associated with lower risk of myocardial infarction when compared with Angio-PCI. However, none of the outcomes were significantly different between IVUS-PCI [RR = 0.88, 95%CI = (0.59, 1.34)], OFDI-PCI [RR = 0.85, 95%CI = (0.23, 3.08)] and Angio-PCI. Detailed results are shown in Table 5.

**Table 5:** League table of myocardial infarction.

Angio-PCI	0.78 (0.62, 0.99)	0.88 (0.59, 1.34)	0.59 (0.35, 0.97)	0.85 (0.23, 3.08)	0.64 (0.45, 0.91)
	FFR-PCI	1.13 (0.74, 1.74)	0.75 (0.43, 1.29)	1.09 (0.29, 3.97)	0.82 (0.54, 1.25)
		IVUS-PCI	0.67 (0.38, 1.16)	0.96 (0.27, 3.22)	0.72 (0.42, 1.24)
			OCT-PCI	1.45 (0.37, 5.51)	1.09 (0.59, 2.05)
				OFDI-PCI	0.75 (0.2, 2.95)
					QFR-PCI

#### 3.4.5 Target vessel revascularization

In 17 trial (9, 10, 14–17, 22–24, 27, 28, 32, 37, 39, 43, 46, 47), the occurrence of TVR was reported. The network meta-analysis showed that the incidence of TVR was significantly lower in patients who underwent IVUS-PCI [RR = 0.57, 95%CI = (0.36, 0.86)], OCT-PCI [RR = 0.47, 95%CI = (0.24, 0.95)] than in those who underwent Angio-PCI. However, CTA-PCI [RR = 1.19, 95%CI = (0.36, 3.87)], FFR-PCI [RR = 0.85, 95%CI = (0.5, 1.47)], OFDI-PCI [RR = 0.47, 95%CI = (0.14, 1.58)], QFR-PCI [RR = 0.74, 95%CI = (0.51, 1.06)] showed no statistically significant difference compared to Angio-PCI. Detailed results are shown in Table 6.

**Table 6:** League table of TVR.

Angio-PCI	1.19 (0.36, 3.87)	0.85 (0.5, 1.47)	0.57 (0.36, 0.86)	0.47 (0.24, 0.95)	0.47 (0.14, 1.58)	0.74 (0.51, 1.06)
	CTA-PCI	0.72 (0.2, 2.55)	0.48 (0.14, 1.66)	0.4 (0.1, 1.55)	0.4 (0.07, 2.06)	0.62 (0.18, 2.11)
		FFR-PCI	0.67 (0.42, 1.03)	0.56 (0.3, 1.06)	0.56 (0.16, 1.87)	0.88 (0.45, 1.64)
			IVUS-PCI	0.84 (0.46, 1.53)	0.84 (0.26, 2.55)	1.31 (0.74, 2.31)
				OCT-PCI	1 (0.27, 3.5)	1.56 (0.71, 3.41)
					OFDI-PCI	1.55 (0.45, 5.64)
						QFR-PCI

### 3.5 Ranking result

**Figure 3:**
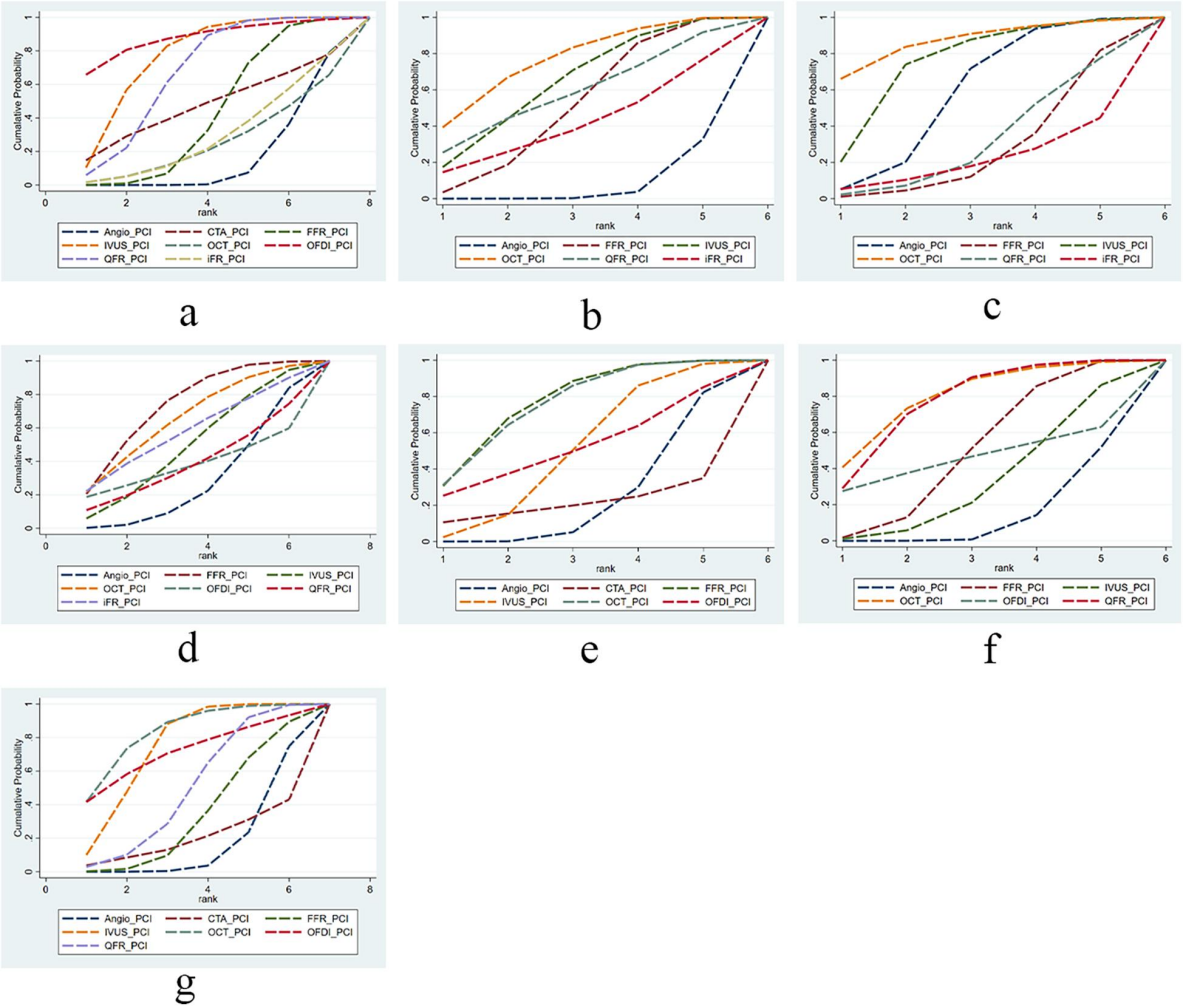
The SUCRA for clinical outcomes among guidance modalities included in the network meta-analysis. **(a)** MACE ≤12 months; **(b)** MACE >12 months; **(c)** all-cause mortality ≤12 months; **(d)** all-cause mortality >12 months; **(e)** cardiac death; **(f)** myocardial infarction; **(g)** TVR.

In this study, we evaluated and ranked the efficacy of PCI with different guidance modalities in reducing cardiovascular events and mortality: OFDI ranked first in reducing short-term MACE; OCT ranked first in reducing long-term MACE, short-term all-cause mortality, incidence of myocardial infarction, and TVR; and FFR ranked first in reducing long-term all-cause mortality and cardiac death. The SUCRA ranking chart visually displays the ranking of the effectiveness of different technologies in each indicator. Figure 3 (Sucra ranking chart).

### 3.6 Publication bias and heterogeneity

We used STATA software to create funnel plots to assess publication bias for the outcome measures included in at least five studies, with different colors showing comparisons between different interventions (Supplementary Figure S4). Deviance Information Criterion (DIC) and node-splitting analysis showed no statistical inconsistencies in the studies (Supplementary S3).

## 4 Discussion

Based on a Bayesian analysis of 40 trials, we evaluated the short-term effects and long-term outcomes of eight different PCI guidance modalities, focusing on the incidence of MACE, all-cause mortality, cardiac death, myocardial infarction, and TVR. Network diagrams and league tables were used to present the comparative efficacy of the different guidance modalities. These results, supported by the network analysis, provide important evidence for clinical practice in selecting the best guidance techniques to improve patient outcomes.

Network meta-analyses of different guidance modalities showed that IVUS significantly reduced the risk of MACE in both the short and long term compared with CAG alone. This result is consistent with the findings of Aylk et al, Park et al, and Yan et al. (48–50), who all emphasized the advantages of IVUS for decision-making orientation and lesion assessment. IVUS enables detailed assessment of the complexity of coronary artery lesions, providing information on vascular anatomy, plaque characteristics, and stent optimization (51). Meanwhile, we found that OFDI and QFR showed the potential to reduce the risk of MACE in the short term, and OFDI ranked first in reducing MACE in the short term. This may be attributed to the fact that OFDI identifies lesions that require intervention through high-resolution imaging (52), while QFR optimizes patient selection through non-invasive hemodynamic analysis and demonstrates great potential in evaluating prognosis (53, 54). In the long term, OCT and FFR also demonstrate the ability to reduce MACE. OCT can provide in-depth lesion analysis and direct measurement of myocardial ischemia to assess the risk of complications (55), while FFR optimizes intervention strategies by accurately measuring hemodynamics (56). These characteristics also help OCT and FFR reduce the incidence of myocardial infarction. Imaging technologies such as IVUS, OFDI, OCT, and FFR play a crucial role in improving the outcomes of PCI treatment in daily clinical practice. IVUS allows for detailed assessment of coronary artery lesions, optimizing stent implantation and reducing the occurrence of MACE. OFDI and QFR help identify patients suitable for intervention through high-resolution imaging and hemodynamic analysis, thus lowering the risk of short-term MACE. OCT provides in-depth lesion analysis and myocardial ischemia assessment, effectively reducing myocardial infarction and complications, particularly in the long term. FFR, by accurately measuring hemodynamics, helps select the most appropriate patients for intervention, further enhancing treatment effectiveness. The application of these imaging technologies significantly improves clinical decision-making, enhances patient prognosis, and reduces the need for re-intervention. Despite the high cost and technical requirements, they offer great potential for personalized treatment of coronary artery disease (57).

Although the Bayesian analysis of all-cause mortality did not show significant differences between the different guidance modalities in either the short or long term, this result is noteworthy. It may reflect the relative consistency of different technologies in reducing all-cause mortality and is consistent with previous research (58, 59). However, the ranking results are notable, with OCT ranking first in the short term (point estimate 0.6) and FFR ranking first in the long term (point estimate 0.58), suggesting that FFR and OCT may have great potential in future evidence-based large sample and multi-center research. Although different intervention techniques (such as FFR, IVUS, OCT, etc.) have their own advantages in reducing specific cardiovascular events (such as MACE or cardiac death), and FFR-PCI in particular significantly reduces cardiac mortality, there seems to be no significant difference in improving overall survival of patients. This may be because these techniques improve the accuracy and safety of the intervention process, but the effect on all-cause mortality is not as significant as expected. In addition, factors such as baseline characteristics, lesion complexity, and comorbidities of patients may also have a significant impact on all-cause mortality, thereby masking potential differences between different techniques. The advantages of IVUS and OCT in reducing the incidence of TVR have been validated and are consistent with the research of Siddiqi TJ (60), while other guidance methods have not shown significant effects. This provides important guidance for clinical practice, recommending that IVUS or OCT be given priority in the interventional treatment of complex coronary artery lesions to optimize patient outcomes and reduce the need for re-intervention. Unlike Siddiqi TJ's study, we ranked the efficacy of OCT and IVUS and found that OCT ranked first in reducing TVR.

It is noteworthy that although different PCI guidance technologies show significant advantages in reducing MACE and TVR, their impact on all-cause mortality did not reach statistical significance. This result may reflect the complex interplay of multiple factors, such as patients' baseline characteristics (e.g., age, comorbidities), lesion complexity, and other interventions during the treatment process. In coronary intervention, the main role of imaging technologies is to optimize intervention strategies, improve intervention precision, and enhance safety. The impact on overall survival may be limited by other more complex factors. Therefore, although imaging technologies help improve the prognosis of specific cardiovascular events, their long-term impact on all-cause mortality needs to be further explored in future studies.

In our network meta-analysis, a total of 40 randomized trials were included, while observational studies were excluded. This approach allowed us to conduct a relatively high-quality evaluation of the existing PCI guidance modalities. However, it is important to acknowledge several limitations. Firstly, although we accounted for heterogeneity in patient baseline characteristics, lesion types, and follow-up durations in our analysis, these factors may still influence the assessment of the efficacy of different guidance methods. Factors such as patient age, comorbidities, clinical presentation (ACS or stable CAD), and lesion complexity could lead to variations in the effectiveness of different techniques in different populations. Additionally, differences in follow-up duration may affect the evaluation of long-term clinical outcomes. Secondly, there is a potential risk of bias in the randomization and allocation processes of the randomized controlled studies included, which may impact the results of this analysis. Moreover, the sample size for some guidance methods is relatively small, particularly for newer technologies like OFDI and QFR, which may affect the stability and credibility of the statistical results. Furthermore, it should be emphasized that in real-world clinical practice, the selection of guidance modalities is not solely determined by lesion complexity or patient characteristics. Practical considerations such as operator experience, equipment availability, reimbursement and billing policies, and institutional resources also play a critical role in decision-making. In many cases, clinicians may choose to combine different modalities to achieve optimal outcomes. Therefore, while our network meta-analysis provides comparative efficacy data, its translation into clinical practice requires individualized decisions that integrate both patient-specific and health system-related factors. Finally, an important future perspective is the potential application of artificial intelligence (AI)-based programs. By analyzing angiographic features and integrating clinical, anatomical, and morphological information, AI tools could assist clinicians in selecting the most appropriate imaging or functional guidance modality, with the aim of reducing MACE during follow-up. Although this approach was beyond the scope of our present analysis, it represents a promising innovation for future individualized PCI strategies.

## 5 Conclusion

In summary, this study is the first to comprehensively evaluate the efficacy of eight different PCI guidance modalities, assessing both short-term and long-term outcomes. The results show that IVUS and OCT are more effective in reducing MACE and TVR, suggesting that IVUS and OCT may be the best strategies for interventions in complex coronary artery lesions. However, while each technology has its advantages in reducing specific events like cardiac death, these advantages are more evident in precisely assessing lesions and optimizing intervention strategies, and their impact on overall survival and mortality requires further validation.

## Data Availability

The original contributions presented in the study are included in the article/Supplementary Material, further inquiries can be directed to the corresponding authors.

## Supplementary material

Supplementary Materials 1 Search strategy.

Supplementary Materials 2 Risk of bias

Supplementary Materials 3 DIC and node-splitting analysis.

Supplementary Materials 4 Funnel plots
