# Supplementary figures and images for: Impact of percutaneous coronary intervention with different guidance modalities in patients with coronary artery lesions: a network meta-analysis and systematic review
Source: Front Cardiovasc Med. 2025 Oct 9;12:1526188. doi: 10.3389/fcvm.2025.1526188 (PMC12546242; doi:10.3389/fcvm.2025.1526188)

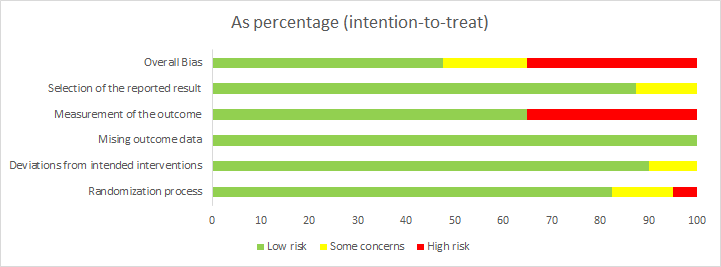

Supplement: Supplementary Materials 2 — Risk of bias [file Datasheet2.zip › Supplementary materials 2/a.png]

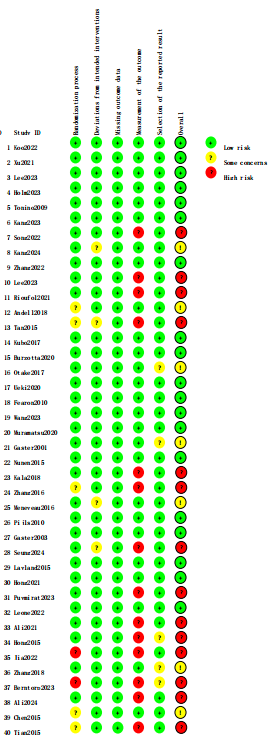

Supplement: Supplementary Materials 2 — Risk of bias [file Datasheet2.zip › Supplementary materials 2/b.png]

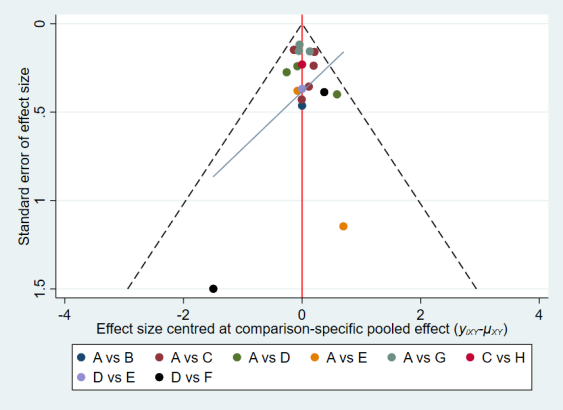

Supplement: Supplementary Materials 4 — Funnel plots [file Datasheet4.zip › Supplementary materials 4/a.tif]

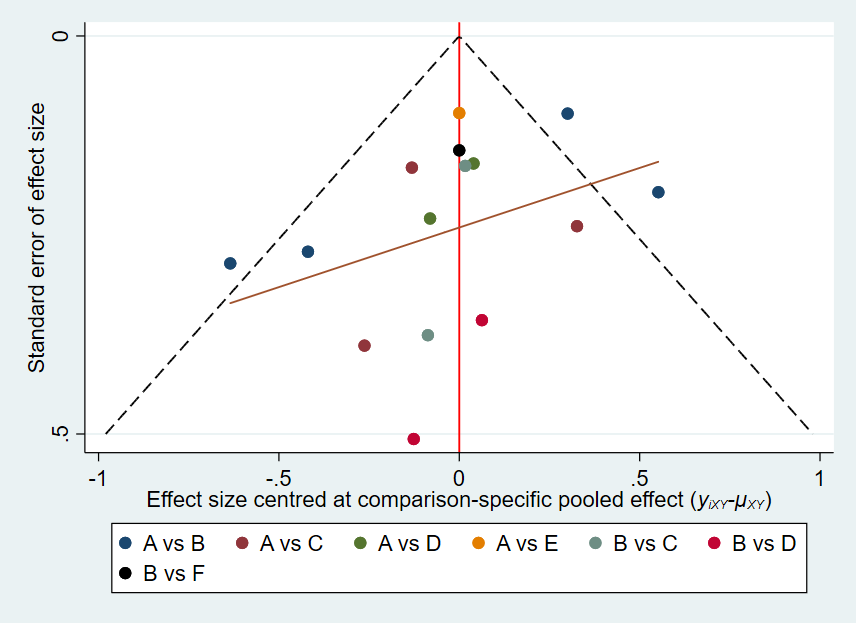

Supplement: Supplementary Materials 4 — Funnel plots [file Datasheet4.zip › Supplementary materials 4/b.tif]

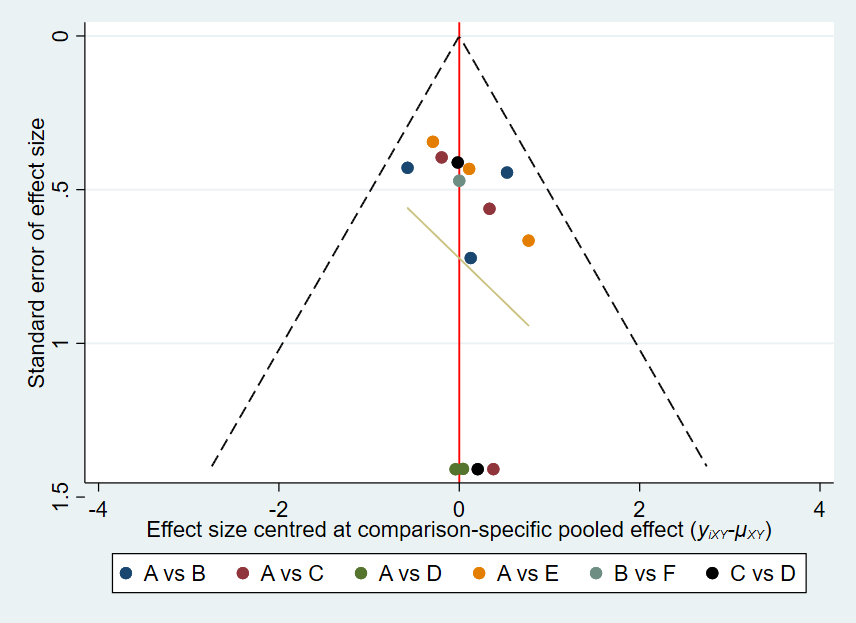

Supplement: Supplementary Materials 4 — Funnel plots [file Datasheet4.zip › Supplementary materials 4/c.tif]

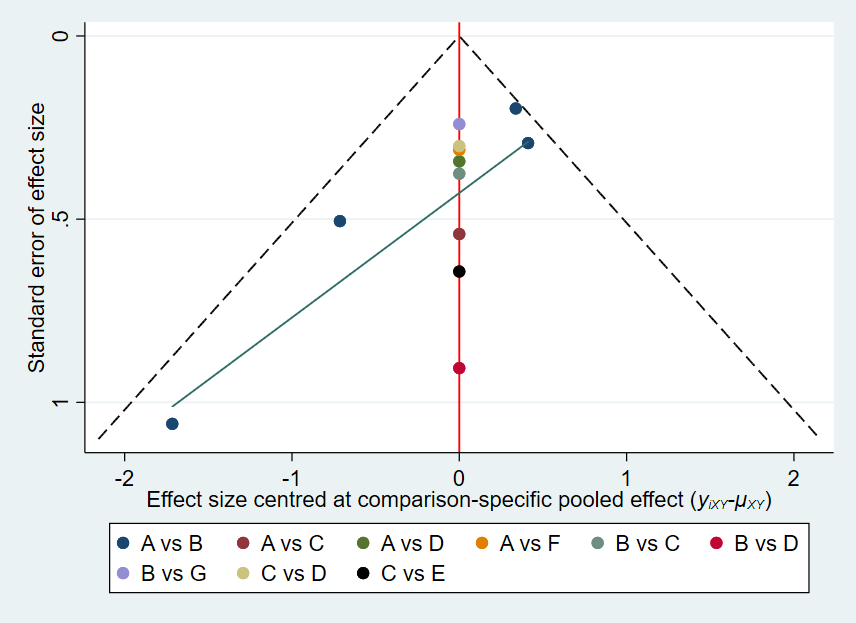

Supplement: Supplementary Materials 4 — Funnel plots [file Datasheet4.zip › Supplementary materials 4/d.tif]

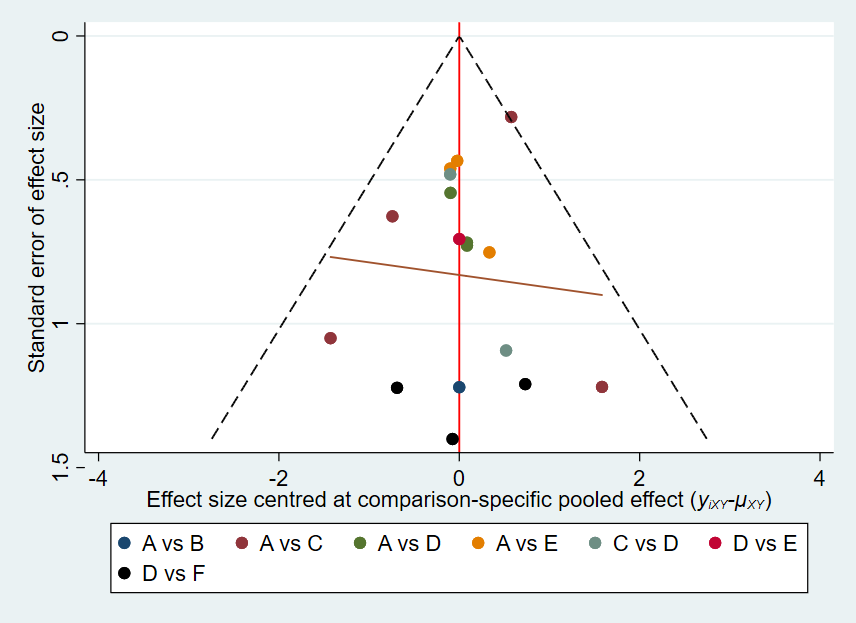

Supplement: Supplementary Materials 4 — Funnel plots [file Datasheet4.zip › Supplementary materials 4/e.tif]

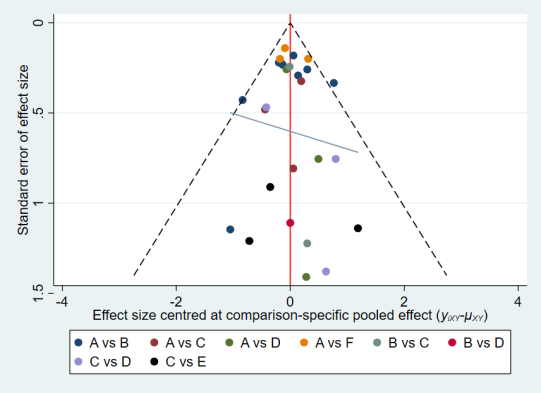

Supplement: Supplementary Materials 4 — Funnel plots [file Datasheet4.zip › Supplementary materials 4/f.tif]

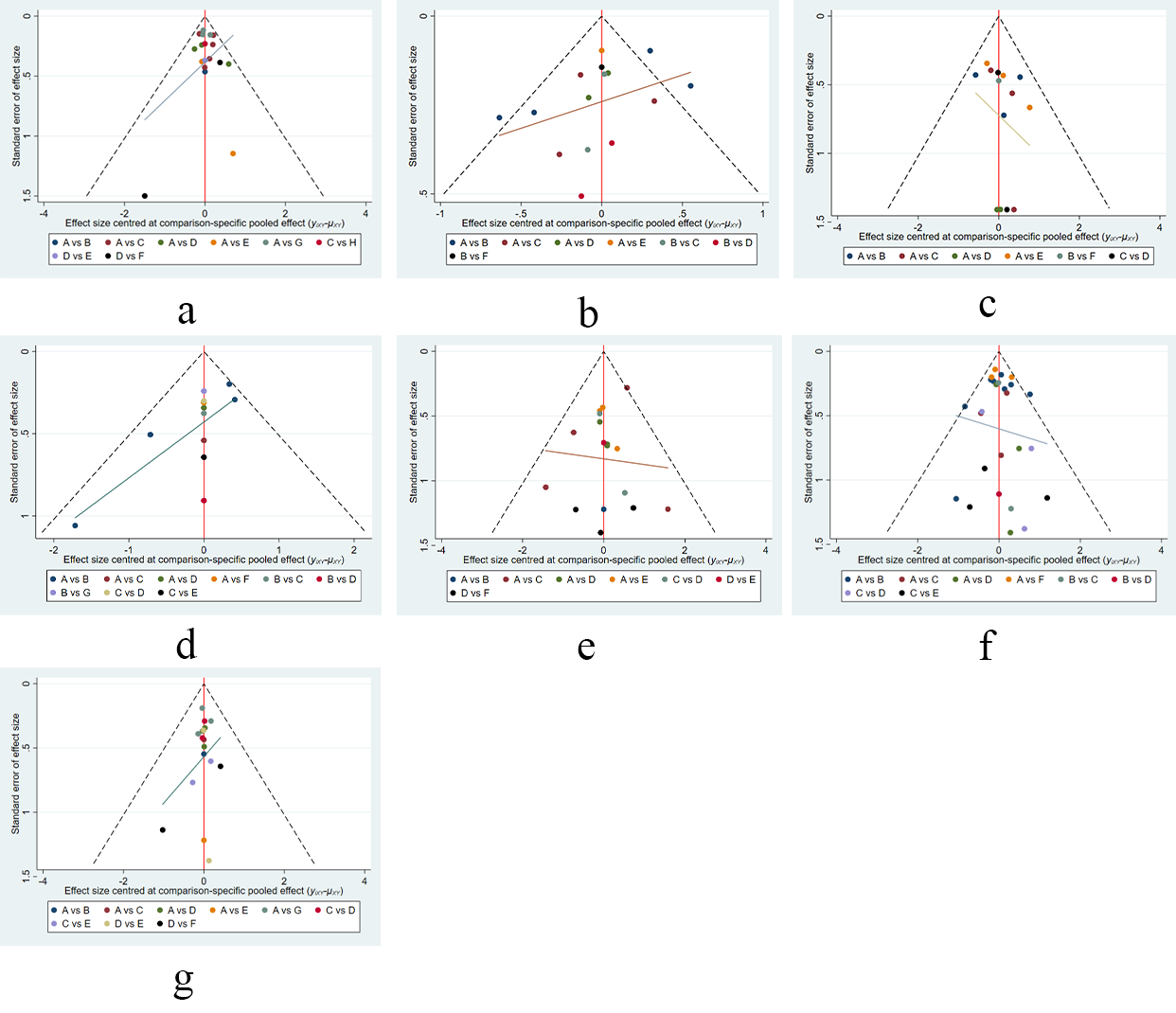

Supplement: Supplementary Materials 4 — Funnel plots [file Datasheet4.zip › Supplementary materials 4/fig4.tif]

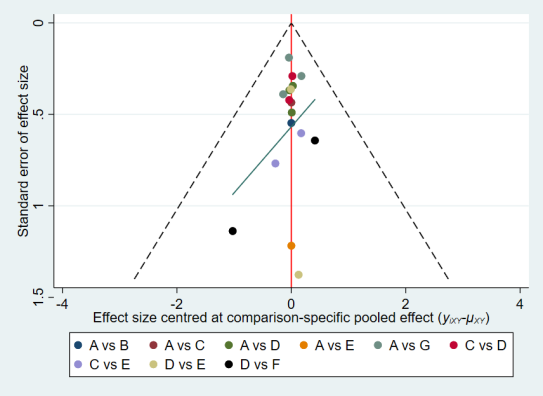

Supplement: Supplementary Materials 4 — Funnel plots [file Datasheet4.zip › Supplementary materials 4/g.tif]
