# Supplementary material for: Impact of percutaneous coronary intervention with different guidance modalities in patients with coronary artery lesions: a network meta-analysis and systematic review
Source: Front Cardiovasc Med. 2025 Oct 9;12:1526188. doi: 10.3389/fcvm.2025.1526188 (PMC12546242; doi:10.3389/fcvm.2025.1526188)

**MACE-RR (≤12)**

**Inconsistency test**

**Results on the Log Risk Ratio scale**

**Iterations = 20001:70000**

**Thinning interval = 1**

**Number of chains = 4**

**Sample size per chain = 50000**

**1. Empirical mean and standard deviation for each variable,**

**plus standard error of the mean:**

**Mean SD Naive SE Time-series SE**

**d.Angio_PCI.CTA_PCI -0.24196 0.50361 0.0011261 0.0096841**

**d.Angio_PCI.FFR_PCI -0.18545 0.10305 0.0002304 0.0010100**

**d.Angio_PCI.IVUS_PCI -0.47516 0.18998 0.0004248 0.0027598**

**d.Angio_PCI.OCT_PCI 0.05979 0.33009 0.0007381 0.0063536**

**d.Angio_PCI.QFR_PCI -0.37184 0.11935 0.0002669 0.0009266**

**d.FFR_PCI.iFR_PCI 0.12315 0.27551 0.0006161 0.0031862**

**d.IVUS_PCI.OFDI_PCI -0.41104 0.40407 0.0009035 0.0081531**

**sd.d 0.11609 0.09309 0.0002082 0.0018697**

**2. Quantiles for each variable:**

**2.5% 25% 50% 75% 97.5%**

**d.Angio_PCI.CTA_PCI -1.266473 -0.57322 -0.23045 0.09797 0.72292**

**d.Angio_PCI.FFR_PCI -0.384017 -0.25017 -0.18705 -0.12276 0.02410**

**d.Angio_PCI.IVUS_PCI -0.846074 -0.60086 -0.47477 -0.34977 -0.09778**

**d.Angio_PCI.OCT_PCI -0.586529 -0.16112 0.05741 0.27440 0.71346**

**d.Angio_PCI.QFR_PCI -0.606966 -0.44402 -0.37199 -0.29929 -0.13639**

**d.FFR_PCI.iFR_PCI -0.418000 -0.05492 0.12492 0.29975 0.66079**

**d.IVUS_PCI.OFDI_PCI -1.215717 -0.67371 -0.40971 -0.14777 0.37274**

**sd.d 0.004568 0.04729 0.09640 0.16109 0.34634**

**-- Model fit (residual deviance):**

**Dbar pD DIC**

**36.11294 26.76026 62.87319**

**35 data points, ratio 1.032, I^2 = 6%**

**consistency test**

**Results on the Log Risk Ratio scale**

**Iterations = 20001:70000**

**Thinning interval = 1**

**Number of chains = 4**

**Sample size per chain = 50000**

**1. Empirical mean and standard deviation for each variable,**

**plus standard error of the mean:**

**Mean SD Naive SE Time-series SE**

**d.Angio_PCI.CTA_PCI -0.22915 0.49933 0.0011165 0.0096259**

**d.Angio_PCI.FFR_PCI -0.18587 0.10309 0.0002305 0.0010442**

**d.Angio_PCI.IVUS_PCI -0.47043 0.18707 0.0004183 0.0027212**

**d.Angio_PCI.OCT_PCI 0.05989 0.32315 0.0007226 0.0059469**

**d.Angio_PCI.QFR_PCI -0.37301 0.11793 0.0002637 0.0008848**

**d.FFR_PCI.iFR_PCI 0.12664 0.27516 0.0006153 0.0032653**

**d.IVUS_PCI.OFDI_PCI -0.39847 0.39600 0.0008855 0.0082845**

**sd.d 0.11385 0.09313 0.0002082 0.0019019**

**2. Quantiles for each variable:**

**2.5% 25% 50% 75% 97.5%**

**d.Angio_PCI.CTA_PCI -1.214797 -0.55684 -0.22452 0.09724 0.75154**

**d.Angio_PCI.FFR_PCI -0.385131 -0.25083 -0.18712 -0.12273 0.02249**

**d.Angio_PCI.IVUS_PCI -0.834917 -0.59427 -0.47214 -0.34866 -0.09909**

**d.Angio_PCI.OCT_PCI -0.580365 -0.15349 0.05874 0.27026 0.69807**

**d.Angio_PCI.QFR_PCI -0.606794 -0.44331 -0.37303 -0.30274 -0.13873**

**d.FFR_PCI.iFR_PCI -0.406091 -0.05149 0.12356 0.30505 0.67330**

**d.IVUS_PCI.OFDI_PCI -1.202810 -0.65373 -0.38816 -0.13261 0.35304**

**sd.d 0.005771 0.04456 0.09254 0.15749 0.35217**

**-- Model fit (residual deviance):**

**Dbar pD DIC**

**35.99736 26.56125 62.55861**

**35 data points, ratio 1.028, I^2 = 6%**

**node-splitting method**


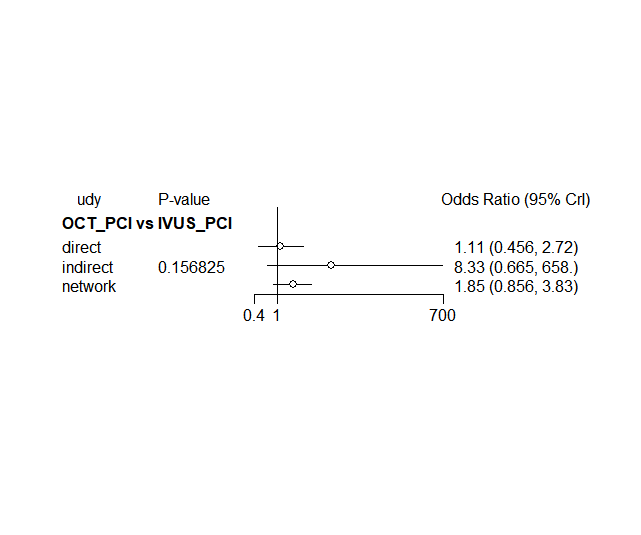


**MACE-RR (＞12m)**

**Inconsistency test**

**Results on the Log Risk Ratio scale**

**Iterations = 20001:70000**

**Thinning interval = 1**

**Number of chains = 4**

**Sample size per chain = 50000**

**1. Empirical mean and standard deviation for each variable,**

**plus standard error of the mean:**

**Mean SD Naive SE Time-series SE**

**d.Angio_PCI.FFR_PCI -0.38393 0.2373 0.0005307 0.0008595**

**d.Angio_PCI.IVUS_PCI -0.48620 0.2843 0.0006356 0.0010257**

**d.Angio_PCI.OCT_PCI -0.39709 0.3262 0.0007293 0.0010393**

**d.Angio_PCI.QFR_PCI -0.38588 0.4283 0.0009578 0.0010627**

**d.FFR_PCI.iFR_PCI 0.10322 0.4428 0.0009902 0.0012891**

**d.FFR_PCI.IVUS_PCI 0.01703 0.3504 0.0007835 0.0014236**

**d.FFR_PCI.OCT_PCI -0.49145 0.4237 0.0009475 0.0022104**

**sd.d 0.38608 0.1622 0.0003626 0.0014556**

**2. Quantiles for each variable:**

**2.5% 25% 50% 75% 97.5%**

**d.Angio_PCI.FFR_PCI -0.8872 -0.5231 -0.37241 -0.2349 0.06548**

**d.Angio_PCI.IVUS_PCI -1.0675 -0.6570 -0.48500 -0.3132 0.08114**

**d.Angio_PCI.OCT_PCI -1.0614 -0.5907 -0.39614 -0.2035 0.25938**

**d.Angio_PCI.QFR_PCI -1.2662 -0.6282 -0.38521 -0.1431 0.49274**

**d.FFR_PCI.iFR_PCI -0.7982 -0.1539 0.10245 0.3605 1.00848**

**d.FFR_PCI.IVUS_PCI -0.6906 -0.1937 0.01829 0.2291 0.71820**

**d.FFR_PCI.OCT_PCI -1.3487 -0.7616 -0.48621 -0.2175 0.33644**

**sd.d 0.1248 0.2738 0.36334 0.4753 0.77642**

**-- Model fit (residual deviance):**

**Dbar pD DIC**

**30.10737 27.65165 57.75902**

**30 data points, ratio 1.004, I^2 = 4%**

**consistency test**

**Results on the Log Risk Ratio scale**

**Iterations = 20001:70000**

**Thinning interval = 1**

**Number of chains = 4**

**Sample size per chain = 50000**

**1. Empirical mean and standard deviation for each variable,**

**plus standard error of the mean:**

**Mean SD Naive SE Time-series SE**

**d.Angio_PCI.FFR_PCI -0.3412 0.1750 0.0003913 0.0007804**

**d.Angio_PCI.IVUS_PCI -0.4193 0.2102 0.0004701 0.0008924**

**d.Angio_PCI.OCT_PCI -0.5436 0.2435 0.0005446 0.0010870**

**d.Angio_PCI.QFR_PCI -0.3892 0.3778 0.0008449 0.0009624**

**d.FFR_PCI.iFR_PCI 0.1037 0.3922 0.0008771 0.0012296**

**sd.d 0.3374 0.1407 0.0003147 0.0012972**

**2. Quantiles for each variable:**

**2.5% 25% 50% 75% 97.5%**

**d.Angio_PCI.FFR_PCI -0.7047 -0.4467 -0.3354 -0.2307 -0.005100**

**d.Angio_PCI.IVUS_PCI -0.8513 -0.5467 -0.4160 -0.2875 -0.006579**

**d.Angio_PCI.OCT_PCI -1.0532 -0.6903 -0.5336 -0.3874 -0.083273**

**d.Angio_PCI.QFR_PCI -1.1623 -0.6041 -0.3893 -0.1747 0.382101**

**d.FFR_PCI.iFR_PCI -0.6922 -0.1259 0.1042 0.3342 0.898382**

**sd.d 0.1110 0.2403 0.3192 0.4140 0.671340**

**-- Model fit (residual deviance):**

**Dbar pD DIC**

**30.30823 26.49662 56.80485**

**30 data points, ratio 1.01, I^2 = 4%**

**node-splitting method**

**
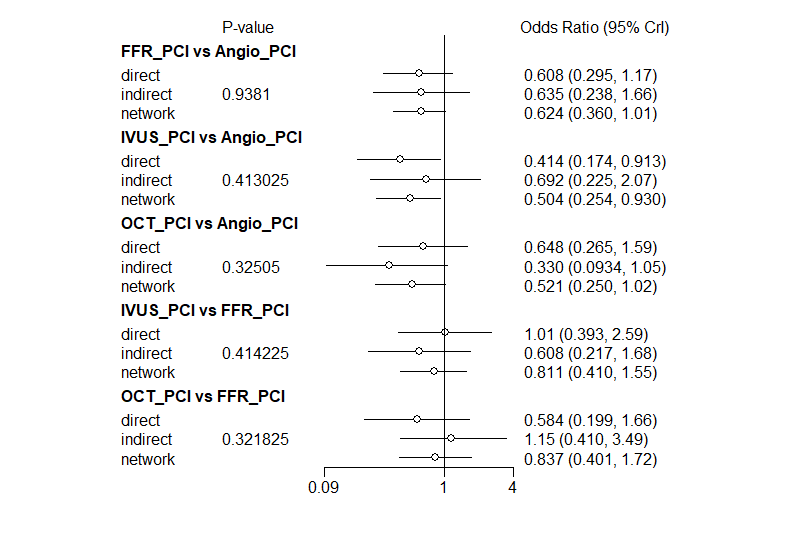
**

**All-cause mortality-RR(≤12m)**

**Inconsistency test**

**Results on the Log Risk Ratio scale**

**Iterations = 20001:70000**

**Thinning interval = 1**

**Number of chains = 4**

**Sample size per chain = 50000**

**1. Empirical mean and standard deviation for each variable,**

**plus standard error of the mean:**

**Mean SD Naive SE Time-series SE**

**d.Angio_PCI.FFR_PCI 0.3934 0.3847 0.0008603 0.003052**

**d.Angio_PCI.IVUS_PCI -0.3086 0.4327 0.0009676 0.003797**

**d.Angio_PCI.OCT_PCI -0.2388 1.0783 0.0024111 0.019370**

**d.Angio_PCI.QFR_PCI 0.3206 0.3641 0.0008142 0.002892**

**d.FFR_PCI.iFR_PCI 0.2163 0.6532 0.0014606 0.005292**

**d.IVUS_PCI.OCT_PCI -0.3487 0.6057 0.0013544 0.003965**

**sd.d 0.3701 0.2313 0.0005172 0.003744**

**2. Quantiles for each variable:**

**2.5% 25% 50% 75% 97.5%**

**d.Angio_PCI.FFR_PCI -0.36543 0.14945 0.3914 0.63449 1.1668**

**d.Angio_PCI.IVUS_PCI -1.16301 -0.58338 -0.3104 -0.03847 0.5618**

**d.Angio_PCI.OCT_PCI -2.52633 -0.91119 -0.2034 0.48209 1.7971**

**d.Angio_PCI.QFR_PCI -0.36523 0.08463 0.3098 0.54060 1.0846**

**d.FFR_PCI.iFR_PCI -1.07773 -0.19792 0.2138 0.62843 1.5267**

**d.IVUS_PCI.OCT_PCI -1.56611 -0.72166 -0.3476 0.03050 0.8665**

**sd.d 0.02091 0.18013 0.3458 0.53422 0.8551**

**-- Model fit (residual deviance):**

**Dbar pD DIC**

**23.16082 20.51797 43.67879**

**25 data points, ratio 0.9264, I^2 = 0%**

**consistency test**

**Results on the Log Risk Ratio scale**

**Iterations = 20001:70000**

**Thinning interval = 1**

**Number of chains = 4**

**Sample size per chain = 50000**

**1. Empirical mean and standard deviation for each variable,**

**plus standard error of the mean:**

**Mean SD Naive SE Time-series SE**

**d.Angio_PCI.FFR_PCI 0.3882 0.3767 0.0008422 0.003021**

**d.Angio_PCI.IVUS_PCI -0.2688 0.4087 0.0009138 0.004443**

**d.Angio_PCI.OCT_PCI -0.5109 0.6094 0.0013627 0.007656**

**d.Angio_PCI.QFR_PCI 0.3200 0.3559 0.0007958 0.002770**

**d.FFR_PCI.iFR_PCI 0.2185 0.6411 0.0014335 0.005129**

**sd.d 0.3530 0.2240 0.0005008 0.003492**

**2. Quantiles for each variable:**

**2.5% 25% 50% 75% 97.5%**

**d.Angio_PCI.FFR_PCI -0.34628 0.1438 0.3837 0.62972 1.1428**

**d.Angio_PCI.IVUS_PCI -1.06813 -0.5346 -0.2751 -0.01186 0.5700**

**d.Angio_PCI.OCT_PCI -1.69572 -0.9068 -0.5124 -0.11953 0.7198**

**d.Angio_PCI.QFR_PCI -0.35998 0.0950 0.3106 0.53350 1.0680**

**d.FFR_PCI.iFR_PCI -1.05276 -0.1893 0.2168 0.62161 1.5099**

**sd.d 0.01968 0.1717 0.3249 0.50603 0.8391**

**-- Model fit (residual deviance):**

**Dbar pD DIC**

**22.45044 19.51892 41.96936**

**25 data points, ratio 0.898, I^2 = 0%**

**node-splitting method**


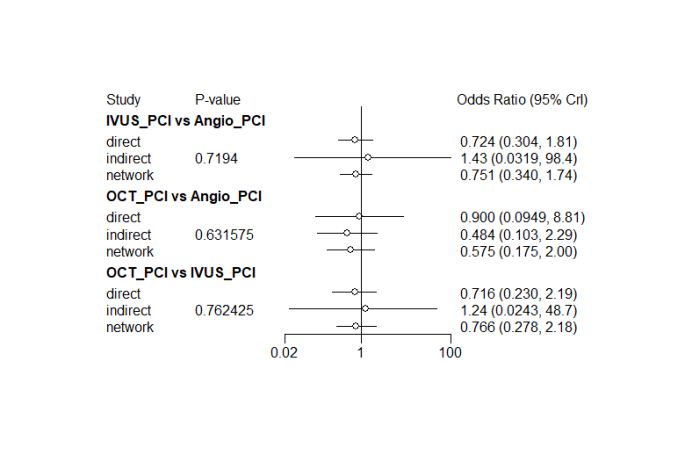


**All-cause mortality-RR (＞12m)**

**Inconsistency test**

**Results on the Log Risk Ratio scale**

**Iterations = 20001:70000**

**Thinning interval = 1**

**Number of chains = 4**

**Sample size per chain = 50000**

**1. Empirical mean and standard deviation for each variable,**

**plus standard error of the mean:**

**Mean SD Naive SE Time-series SE**

**d.Angio_PCI.FFR_PCI -0.68244 0.5737 0.001283 0.002784**

**d.Angio_PCI.IVUS_PCI -0.16291 1.1458 0.002562 0.004404**

**d.Angio_PCI.OCT_PCI -0.58323 1.0599 0.002370 0.002997**

**d.Angio_PCI.QFR_PCI -0.04871 1.0504 0.002349 0.002996**

**d.FFR_PCI.iFR_PCI 0.13405 1.0275 0.002298 0.002669**

**d.FFR_PCI.IVUS_PCI 0.55954 1.0727 0.002399 0.003789**

**d.FFR_PCI.OCT_PCI -0.48324 1.4322 0.003202 0.008855**

**d.IVUS_PCI.OCT_PCI 0.09944 1.0448 0.002336 0.002898**

**d.IVUS_PCI.OFDI_PCI 0.25801 1.2148 0.002716 0.005934**

**sd.d 0.89198 0.4603 0.001029 0.005620**

**2. Quantiles for each variable:**

**2.5% 25% 50% 75% 97.5%**

**d.Angio_PCI.FFR_PCI -1.98503 -0.99521 -0.61932 -0.326586 0.361**

**d.Angio_PCI.IVUS_PCI -2.53124 -0.82886 -0.15756 0.503495 2.199**

**d.Angio_PCI.OCT_PCI -2.81275 -1.15760 -0.58591 -0.009108 1.633**

**d.Angio_PCI.QFR_PCI -2.26887 -0.60923 -0.05066 0.514211 2.172**

**d.FFR_PCI.iFR_PCI -2.04174 -0.39816 0.13600 0.666383 2.312**

**d.FFR_PCI.IVUS_PCI -1.68377 -0.03023 0.55805 1.149552 2.799**

**d.FFR_PCI.OCT_PCI -3.41491 -1.36856 -0.46429 0.432884 2.335**

**d.IVUS_PCI.OCT_PCI -2.10405 -0.45471 0.09695 0.656470 2.301**

**d.IVUS_PCI.OFDI_PCI -2.22426 -0.46829 0.25107 0.986063 2.745**

**sd.d 0.09163 0.53332 0.85989 1.242493 1.766**

**-- Model fit (residual deviance):**

**Dbar pD DIC**

**25.16840 23.80125 48.96965**

**24 data points, ratio 1.049, I^2 = 9%**

**consistency test**

**Results on the Log Risk Ratio scale**

**Iterations = 20001:70000**

**Thinning interval = 1**

**Number of chains = 4**

**Sample size per chain = 50000**

**1. Empirical mean and standard deviation for each variable,**

**plus standard error of the mean:**

**Mean SD Naive SE Time-series SE**

**d.Angio_PCI.FFR_PCI -0.57673 0.3726 0.0008332 0.002297**

**d.Angio_PCI.IVUS_PCI -0.27733 0.5306 0.0011865 0.002780**

**d.Angio_PCI.OCT_PCI -0.46718 0.5438 0.0012161 0.002926**

**d.Angio_PCI.QFR_PCI -0.05218 0.7413 0.0016575 0.002663**

**d.FFR_PCI.iFR_PCI 0.13516 0.7145 0.0015976 0.002288**

**d.IVUS_PCI.OFDI_PCI 0.26262 0.9558 0.0021373 0.006620**

**sd.d 0.57139 0.3565 0.0007972 0.004483**

**2. Quantiles for each variable:**

**2.5% 25% 50% 75% 97.5%**

**d.Angio_PCI.FFR_PCI -1.42572 -0.7738 -0.53693 -0.34523 0.0698**

**d.Angio_PCI.IVUS_PCI -1.40834 -0.5671 -0.25942 0.03201 0.7516**

**d.Angio_PCI.OCT_PCI -1.66029 -0.7565 -0.43917 -0.14577 0.5548**

**d.Angio_PCI.QFR_PCI -1.57691 -0.4478 -0.05462 0.34278 1.4910**

**d.FFR_PCI.iFR_PCI -1.35049 -0.2300 0.13439 0.49654 1.6268**

**d.IVUS_PCI.OFDI_PCI -1.63134 -0.3318 0.25557 0.85021 2.1780**

**sd.d 0.05287 0.3131 0.50694 0.76209 1.4736**

**-- Model fit (residual deviance):**

**Dbar pD DIC**

**24.95693 21.84623 46.80316**

**24 data points, ratio 1.04, I^2 = 8%**

**node-splitting method**


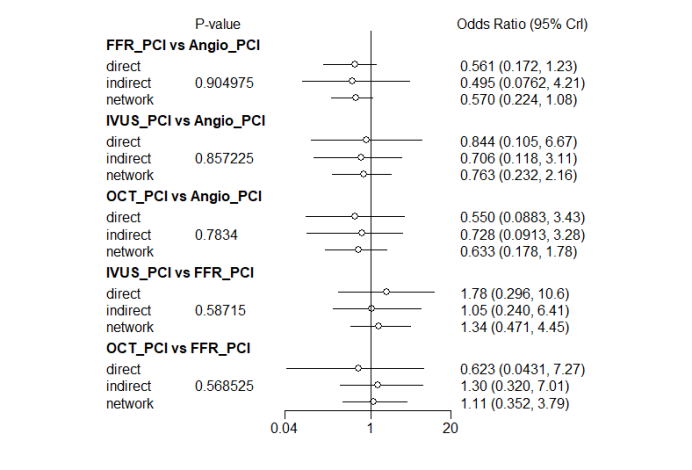

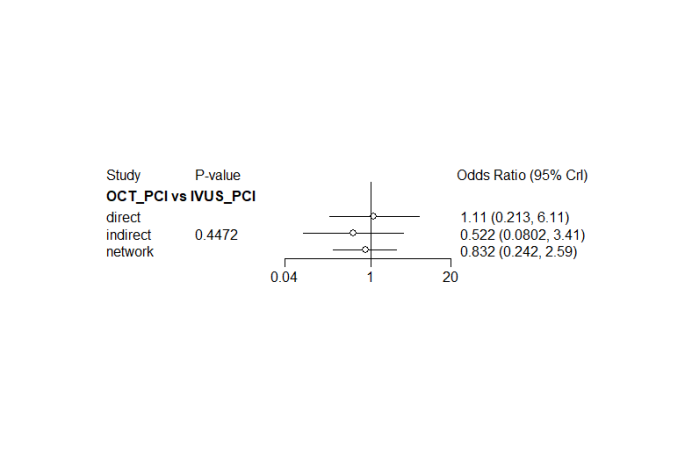


**cardiac death-RR**

**Inconsistency test**

**Results on the Log Risk Ratio scale**

**Iterations = 20001:70000**

**Thinning interval = 1**

**Number of chains = 4**

**Sample size per chain = 50000**

**1. Empirical mean and standard deviation for each variable,**

**plus standard error of the mean:**

**Mean SD Naive SE Time-series SE**

**d.Angio_PCI.CTA_PCI 0.95365 1.5852 0.0035445 0.027959**

**d.Angio_PCI.FFR_PCI -0.86302 0.4149 0.0009277 0.003158**

**d.Angio_PCI.IVUS_PCI -0.62001 0.5011 0.0011204 0.003892**

**d.Angio_PCI.OCT_PCI -0.60501 0.4441 0.0009930 0.002924**

**d.FFR_PCI.IVUS_PCI 0.65719 0.6249 0.0013972 0.005669**

**d.IVUS_PCI.OCT_PCI -0.77593 0.9414 0.0021051 0.008686**

**d.IVUS_PCI.OFDI_PCI 0.01939 0.8123 0.0018163 0.011031**

**sd.d 0.45537 0.3153 0.0007049 0.005306**

**2. Quantiles for each variable:**

**2.5% 25% 50% 75% 97.5%**

**d.Angio_PCI.CTA_PCI -1.93878 -0.09199 0.84723 1.8797 4.4613**

**d.Angio_PCI.FFR_PCI -1.77800 -1.09189 -0.83226 -0.6023 -0.1157**

**d.Angio_PCI.IVUS_PCI -1.63126 -0.93266 -0.61399 -0.3020 0.3588**

**d.Angio_PCI.OCT_PCI -1.49111 -0.87268 -0.60369 -0.3374 0.2852**

**d.FFR_PCI.IVUS_PCI -0.50395 0.25047 0.63283 1.0283 1.9740**

**d.IVUS_PCI.OCT_PCI -2.70361 -1.36948 -0.74977 -0.1643 1.0141**

**d.IVUS_PCI.OFDI_PCI -1.60045 -0.51003 0.02793 0.5497 1.6240**

**sd.d 0.02261 0.21979 0.40428 0.6260 1.2281**

**-- Model fit (residual deviance):**

**Dbar pD DIC**

**33.09911 27.82091 60.92002**

**34 data points, ratio 0.9735, I^2 = 0.3%**

**consistency test**

**Results on the Log Risk Ratio scale**

**Iterations = 20001:70000**

**Thinning interval = 1**

**Number of chains = 4**

**Sample size per chain = 50000**

**1. Empirical mean and standard deviation for each variable,**

**plus standard error of the mean:**

**Mean SD Naive SE Time-series SE**

**d.Angio_PCI.CTA_PCI 0.99910 1.5702 0.0035110 0.032351**

**d.Angio_PCI.FFR_PCI -0.88673 0.3291 0.0007360 0.003161**

**d.Angio_PCI.IVUS_PCI -0.39267 0.3464 0.0007745 0.003363**

**d.Angio_PCI.OCT_PCI -0.69513 0.3667 0.0008199 0.003209**

**d.IVUS_PCI.OFDI_PCI -0.02693 0.8013 0.0017918 0.013456**

**sd.d 0.36215 0.2598 0.0005808 0.004583**

**2. Quantiles for each variable:**

**2.5% 25% 50% 75% 97.5%**

**d.Angio_PCI.CTA_PCI -1.84300 -0.03695 0.89950 1.9176 4.42492**

**d.Angio_PCI.FFR_PCI -1.60560 -1.07706 -0.86621 -0.6720 -0.29383**

**d.Angio_PCI.IVUS_PCI -1.09223 -0.61095 -0.38822 -0.1730 0.28738**

**d.Angio_PCI.OCT_PCI -1.43234 -0.92309 -0.68991 -0.4649 0.01716**

**d.IVUS_PCI.OFDI_PCI -1.65277 -0.54808 -0.01096 0.5014 1.51995**

**sd.d 0.01912 0.16631 0.31827 0.5016 0.99230**

**-- Model fit (residual deviance):**

**Dbar pD DIC**

**32.35627 25.71774 58.07401**

**34 data points, ratio 0.9517, I^2 = 0%**

**node-splitting method**


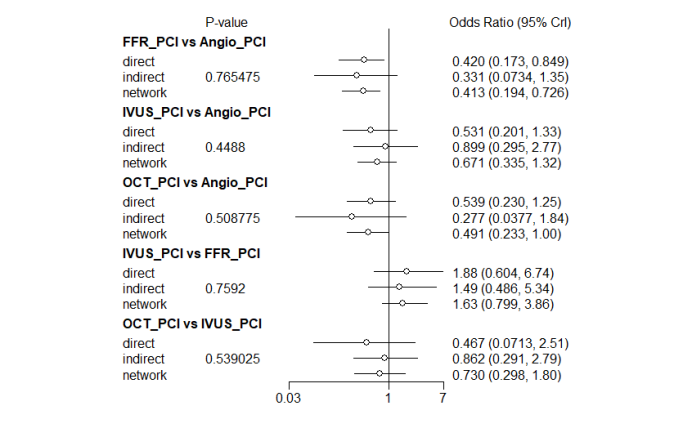


**TVR**

**Inconsistency test**

**Results on the Log Risk Ratio scale**

**Iterations = 20001:70000**

**Thinning interval = 1**

**Number of chains = 4**

**Sample size per chain = 50000**

**1. Empirical mean and standard deviation for each variable,**

**plus standard error of the mean:**

**Mean SD Naive SE Time-series SE**

**d.Angio_PCI.CTA_PCI 0.1545 0.6032 0.0013489 0.010555**

**d.Angio_PCI.FFR_PCI -0.2120 0.5008 0.0011198 0.007991**

**d.Angio_PCI.IVUS_PCI -0.6251 0.2561 0.0005727 0.003553**

**d.Angio_PCI.OCT_PCI 0.8823 1.3965 0.0031226 0.050574**

**d.Angio_PCI.QFR_PCI -0.3030 0.1972 0.0004409 0.001962**

**d.FFR_PCI.IVUS_PCI -0.3323 0.2856 0.0006386 0.003539**

**d.FFR_PCI.OCT_PCI -0.9341 0.5162 0.0011544 0.011101**

**d.IVUS_PCI.OCT_PCI -0.1443 0.4079 0.0009120 0.006774**

**d.IVUS_PCI.OFDI_PCI -0.1918 0.5752 0.0012861 0.012979**

**sd.d 0.1591 0.1388 0.0003104 0.002888**

**2. Quantiles for each variable:**

**2.5% 25% 50% 75% 97.5%**

**d.Angio_PCI.CTA_PCI -1.0113 -0.24836 0.1470 0.5531 1.34505**

**d.Angio_PCI.FFR_PCI -1.1905 -0.54298 -0.2081 0.1208 0.74742**

**d.Angio_PCI.IVUS_PCI -1.1373 -0.78934 -0.6265 -0.4560 -0.12492**

**d.Angio_PCI.OCT_PCI -1.7252 -0.06489 0.8043 1.7508 3.89657**

**d.Angio_PCI.QFR_PCI -0.6958 -0.42467 -0.3007 -0.1818 0.08791**

**d.FFR_PCI.IVUS_PCI -0.8939 -0.51850 -0.3311 -0.1461 0.22365**

**d.FFR_PCI.OCT_PCI -2.0238 -1.27074 -0.9088 -0.5763 0.01243**

**d.IVUS_PCI.OCT_PCI -0.9584 -0.41049 -0.1419 0.1203 0.65660**

**d.IVUS_PCI.OFDI_PCI -1.3489 -0.57006 -0.1796 0.1920 0.91698**

**sd.d 0.0063 0.05566 0.1226 0.2226 0.52579**

**-- Model fit (residual deviance):**

**Dbar pD DIC**

**29.40213 27.61678 57.01890**

**34 data points, ratio 0.8648, I^2 = 0%**

**consistency test**

**Results on the Log Risk Ratio scale**

**Iterations = 20001:70000**

**Thinning interval = 1**

**Number of chains = 4**

**Sample size per chain = 50000**

**1. Empirical mean and standard deviation for each variable,**

**plus standard error of the mean:**

**Mean SD Naive SE Time-series SE**

**d.Angio_PCI.CTA_PCI 0.1707 0.5976 0.0013362 0.011745**

**d.Angio_PCI.FFR_PCI -0.1633 0.2730 0.0006103 0.005112**

**d.Angio_PCI.IVUS_PCI -0.5704 0.2214 0.0004952 0.004059**

**d.Angio_PCI.OCT_PCI -0.7420 0.3567 0.0007976 0.007873**

**d.Angio_PCI.QFR_PCI -0.3025 0.1864 0.0004168 0.001982**

**d.IVUS_PCI.OFDI_PCI -0.1785 0.5840 0.0013059 0.015108**

**sd.d 0.1394 0.1178 0.0002634 0.002367**

**2. Quantiles for each variable:**

**2.5% 25% 50% 75% 97.5%**

**d.Angio_PCI.CTA_PCI -1.008111 -0.22935 0.1714 0.56659 1.35210**

**d.Angio_PCI.FFR_PCI -0.691371 -0.34334 -0.1664 0.01383 0.38862**

**d.Angio_PCI.IVUS_PCI -1.013133 -0.71851 -0.5695 -0.41923 -0.14509**

**d.Angio_PCI.OCT_PCI -1.438173 -0.97762 -0.7449 -0.50153 -0.04859**

**d.Angio_PCI.QFR_PCI -0.671635 -0.41996 -0.3018 -0.18332 0.05991**

**d.IVUS_PCI.OFDI_PCI -1.343422 -0.56319 -0.1728 0.20990 0.93605**

**sd.d 0.004941 0.05155 0.1107 0.19500 0.43775**

**-- Model fit (residual deviance):**

**Dbar pD DIC**

**28.39997 24.64361 53.04359**

**34 data points, ratio 0.8353, I^2 = 0%**

**node-splitting method**


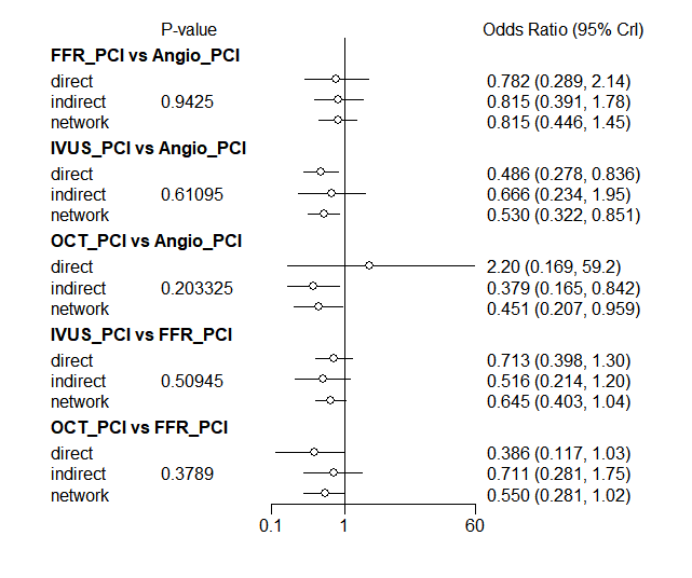

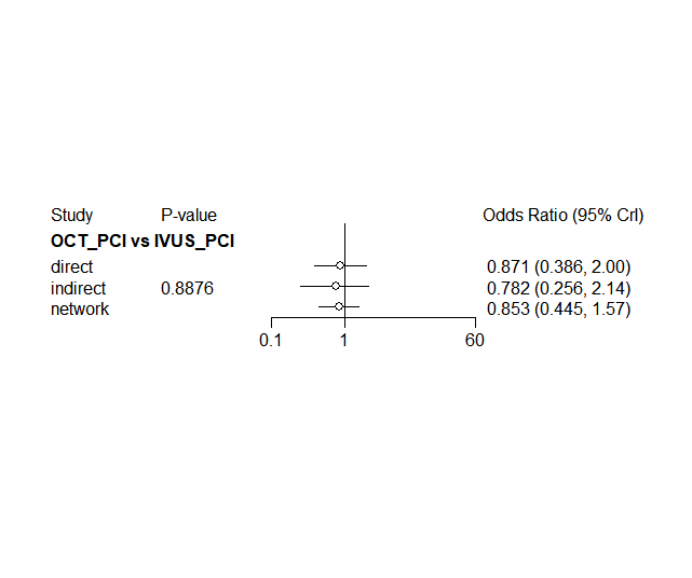
**myocardial infarction**

**Inconsistency test**

**Results on the Log Risk Ratio scale**

**Iterations = 20001:70000**

**Thinning interval = 1**

**Number of chains = 4**

**Sample size per chain = 50000**

**1. Empirical mean and standard deviation for each variable,**

**plus standard error of the mean:**

**Mean SD Naive SE Time-series SE**

**d.Angio_PCI.FFR_PCI -0.29489 0.1228 0.0002746 0.0010350**

**d.Angio_PCI.IVUS_PCI -0.06858 0.2885 0.0006451 0.0033768**

**d.Angio_PCI.OCT_PCI -0.23824 0.3050 0.0006821 0.0035802**

**d.Angio_PCI.QFR_PCI -0.45009 0.1717 0.0003840 0.0009433**

**d.FFR_PCI.IVUS_PCI -0.14171 0.3269 0.0007310 0.0032372**

**d.FFR_PCI.OCT_PCI -1.43128 1.3789 0.0030832 0.0383671**

**d.IVUS_PCI.OCT_PCI -0.97400 0.5080 0.0011358 0.0081070**

**d.IVUS_PCI.OFDI_PCI -0.01581 0.6035 0.0013495 0.0128165**

**sd.d 0.19735 0.1295 0.0002896 0.0026921**

**2. Quantiles for each variable:**

**2.5% 25% 50% 75% 97.5%**

**d.Angio_PCI.FFR_PCI -0.54356 -0.37143 -0.293393 -0.21657 -0.05600**

**d.Angio_PCI.IVUS_PCI -0.64038 -0.25686 -0.065623 0.12085 0.49625**

**d.Angio_PCI.OCT_PCI -0.83823 -0.43551 -0.241632 -0.04113 0.37058**

**d.Angio_PCI.QFR_PCI -0.79048 -0.55204 -0.451735 -0.35024 -0.09868**

**d.FFR_PCI.IVUS_PCI -0.78987 -0.34852 -0.141833 0.06588 0.50880**

**d.FFR_PCI.OCT_PCI -4.60507 -2.18560 -1.284035 -0.47870 0.82144**

**d.IVUS_PCI.OCT_PCI -2.00671 -1.30270 -0.964984 -0.63123 -0.01991**

**d.IVUS_PCI.OFDI_PCI -1.21847 -0.41406 -0.007283 0.38929 1.15995**

**sd.d 0.01273 0.09901 0.180859 0.27296 0.49692**

**-- Model fit (residual deviance):**

**Dbar pD DIC**

**50.88716 38.58632 89.47349**

**51 data points, ratio 0.9978, I^2 = 2%**

**consistency test**

**Results on the Log Risk Ratio scale**

**Iterations = 20001:70000**

**Thinning interval = 1**

**Number of chains = 4**

**Sample size per chain = 50000**

**1. Empirical mean and standard deviation for each variable,**

**plus standard error of the mean:**

**Mean SD Naive SE Time-series SE**

**d.Angio_PCI.FFR_PCI -0.24923 0.1176 0.0002630 0.0009573**

**d.Angio_PCI.IVUS_PCI -0.12621 0.2122 0.0004745 0.0024758**

**d.Angio_PCI.OCT_PCI -0.53433 0.2603 0.0005820 0.0032250**

**d.Angio_PCI.QFR_PCI -0.45029 0.1738 0.0003887 0.0008906**

**d.IVUS_PCI.OFDI_PCI -0.03571 0.6296 0.0014079 0.0135364**

**sd.d 0.20700 0.1250 0.0002796 0.0025626**

**2. Quantiles for each variable:**

**2.5% 25% 50% 75% 97.5%**

**d.Angio_PCI.FFR_PCI -0.48275 -0.3229 -0.24931 -0.17614 -0.01338**

**d.Angio_PCI.IVUS_PCI -0.53557 -0.2666 -0.12878 0.01345 0.29604**

**d.Angio_PCI.OCT_PCI -1.05777 -0.7032 -0.53099 -0.36324 -0.02547**

**d.Angio_PCI.QFR_PCI -0.79620 -0.5548 -0.45282 -0.34869 -0.09140**

**d.IVUS_PCI.OFDI_PCI -1.29156 -0.4571 -0.03581 0.39357 1.17016**

**sd.d 0.01452 0.1121 0.19634 0.28484 0.48421**

**-- Model fit (residual deviance):**

**Dbar pD DIC**

**51.06981 36.36933 87.43914**

**51 data points, ratio 1.001, I^2 = 2%**

**node-splitting method**


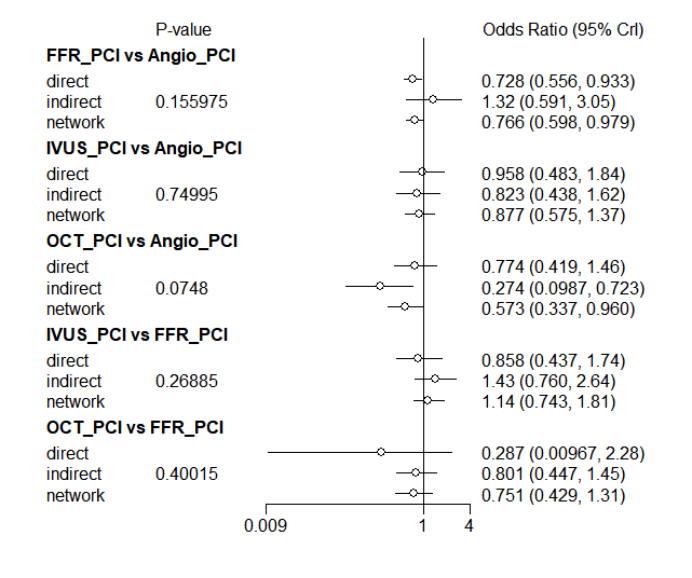

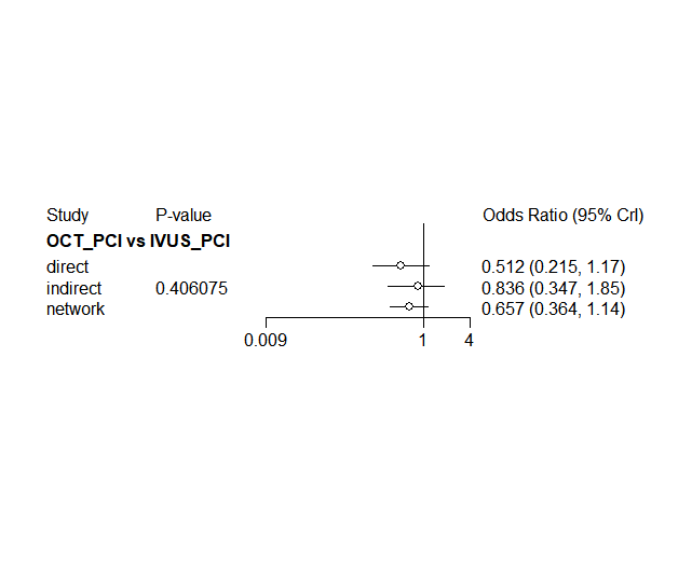

Supplement: Supplementary Materials 3 — DIC and node-splitting analysis. [file Datasheet3.zip › Supplementary materials 3/Supplementary materials 3.docx]
